# Development and Characterization of Shikonin-Loaded NLC with Enhanced Photostability in Hydrogel-Based Dermal Patch

**DOI:** 10.3390/pharmaceutics18101270

**Published:** 2026-10-07

**Authors:** Kittithat Rueangpraphai, Kanokwan Kiattisin, Taepin Junmahasathien

**Affiliations:** Department of Pharmaceutical Sciences, Faculty of Pharmacy, Chiang Mai University, Chiang Mai 50200, Thailand; kittithat_ru@cmu.ac.th (K.R.); kanokwan.k@cmu.ac.th (K.K.)

**Keywords:** shikonin, hydrogel, dermal patch, dermal delivery system, nanostructured lipid carriers, photostability, membrane retention

## Abstract

**Background/Objectives:** Shikonin is a bioactive compound with promising pharmaceutical potential, particularly for anti-inflammation and ability to inhibit proliferative scar formation. However, its applications are limited by poor photostability. Therefore, this research aimed to develop and optimize shikonin-loaded NLC to improve the photostability of shikonin. The optimized NLC formulation was subsequently incorporated into a hydrogel matrix to fabricate a dermal patch, providing a potential alternative for topical scar treatment and self-care application. **Methods:** Shikonin-loaded NLCs were developed, optimized, and evaluated for physicochemical properties, entrapment efficiency, photostability, and in vitro release. The optimized NLC was incorporated into a hyaluronic acid–carboxymethyl cellulose hydrogel matrix to fabricate and evaluate an NLC-based dermal patch. **Results:** The optimized shikonin-loaded NLC exhibited a particle size of 104.94 nm with a narrow polydispersity index of 0.15, and a zeta potential of −34.34 ± 0.02 mV. The NLC achieved high entrapment efficiency (>90%). The NLC significantly enhanced the photostability of shikonin, by increasing the percentage of shikonin remaining after light exposure from 37.36 to 64.55% (*p* < 0.05). In addition, the cumulative shikonin release over 12 h was 15.61 ± 4.18%, following Korsmeyer–Peppas model. A combination of hyaluronic acid and carboxymethyl cellulose (1% *w*/*w*) was employed as the biopolymer for the hydrogel dermal patch. The shikonin-loaded NLC-based dermal patch exhibited favorable physicochemical characteristics. **Conclusions:** The NLC demonstrated enhanced photostability and release properties, which are preserved upon their integration into the hydrogel. The incorporation of shikonin-loaded NLC into a hydrogel matrix provides a viable strategy for stabilizing this light-sensitive bioactive compound. Consequently, this NLC-loaded hydrogel system represents a promising platform for future topical dermal delivery in pharmaceutical and cosmetic applications.

## 1. Introduction

**Figure 1:**
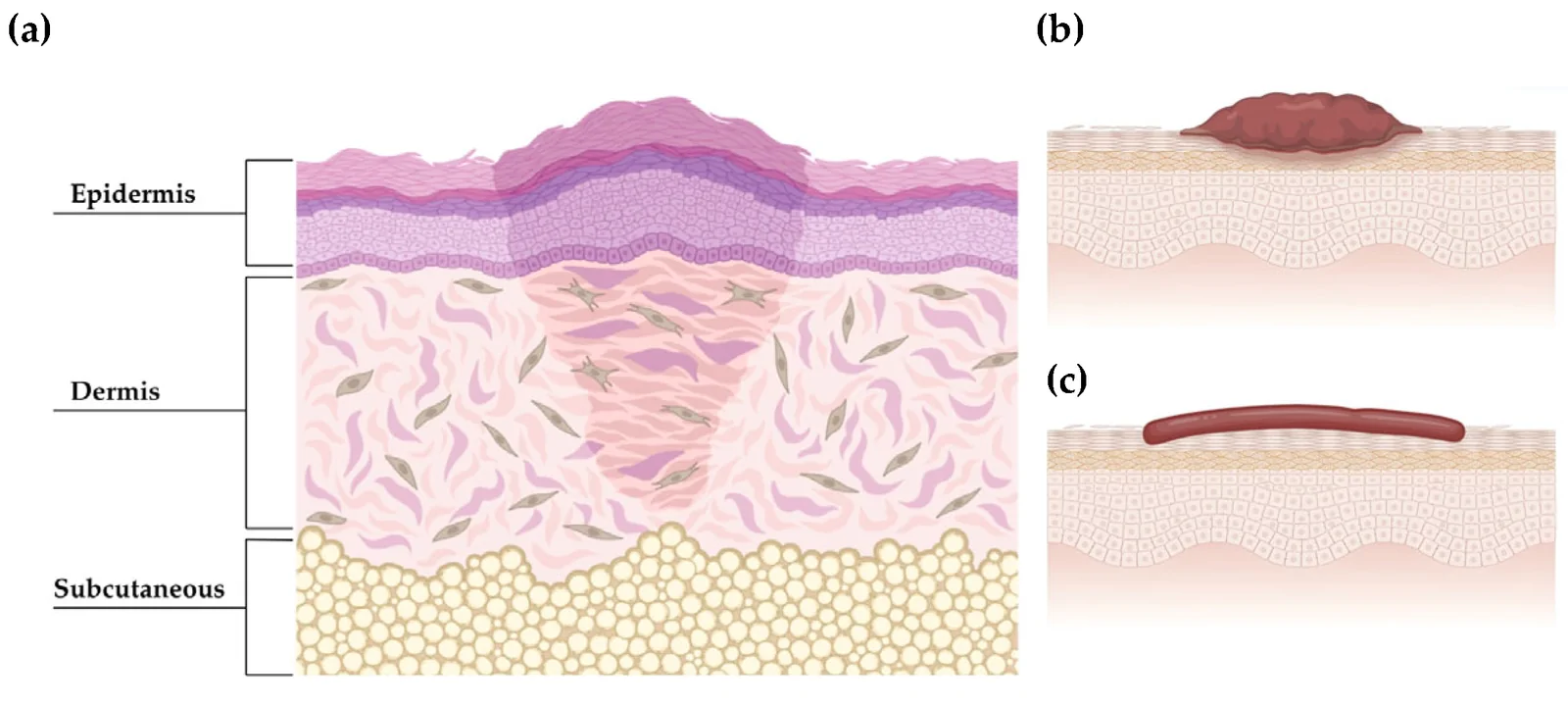
Skin structure and proliferative scars (**a**); keloid (**b**), and hypertrophic scar (**c**), from BioRender.

Cutaneous scars represent the physiological outcome of the wound healing process. Cutaneous pathological scars can be divided into three types, including normotrophic scar, proliferative scar (hypertrophic scar and keloids), and atrophic scar. Wound healing is a complex and dynamic biological process that occurs through four overlapping phases: hemostasis phase, inflammatory phase, proliferation phase, and remodeling phase [1]. Dysregulation of these processes may contribute to abnormal scar formation. Proliferative scar, as shown in Figure 1, are fibroproliferative disorders characterized by excessive deposition of extracellular matrix (ECM) components and persistent activation of fibroblasts [2]. These lesions are among the most common forms of abnormal scarring and can cause significant cosmetic and psychological concerns. Hypertrophic scar is the result of excessive proliferation of ECM and increased collagen deposition within the confines of the original wound. In contrast, keloids extend beyond the original wound boundaries and invade the surrounding normal skin [3]. The development of pathological scars is associated with abnormalities during the remodeling phase of wound healing. During the normal remodeling phase, myofibroblasts responsible for wound contraction undergo apoptosis once tissue repair is completed. However, impaired apoptosis leads to the persistent accumulation of myofibroblasts, contributing to excessive ECM production and scar elevation. Moreover, the excessive accumulation of immature collagen has been recognized as a major contributor to the pathogenesis of hypertrophic scars and keloids [4].

Currently, several scar treatment modalities are available to reduce scar formation and improve skin appearance, including pharmacological therapies, laser-based procedures, and surgical approaches. However, these treatments are often associated with high cost, prolonged treatment period, and variable therapeutic outcomes. In addition, long-term use of conventional treatments may lead to undesirable adverse effects, such as skin atrophy, hyperpigmentation, infection, pain, swelling, and erythema. Natural extracts and their bioactive compounds have gained increasing attention as alternative therapeutic agents in dermatology due to their diverse pharmacological properties and favorable safety profiles. Among these, shikonin is a naturally red naphthoquinone extracted from the roots of *Lithospermum erythrorhizon* [5]. In recent decades, it has been widely used in traditional Chinese herbal formulations and developed into topical preparations for the treatment of skin diseases [5,6]. The pharmacological properties of shikonin have been studied quite extensively in recent years, including antioxidants, anti-inflammatory, antibacterial, and anti-tumor activities [7,8,9,10,11]. Additionally, it has been reported to inhibit proliferative scar formation through multiple mechanisms. Previous studies have demonstrated that it inhibited the expression of cytokeratin 10, transforming growth factor β1, α-smooth muscle actin, and type I collagen in scar formation [12]. It has induced an apoptotic effect on human hypertrophic scar fibroblasts, as evidenced by morphological changes and Terminal deoxynucleotidyl transferase dUTP nick end labeling assay results, at an effective concentration of 1 μg/mL [13,14]. It selectively inhibits fibroblast proliferation while preserving keratinocyte viability and function, suggesting a favorable therapeutic profile for scar management. Its pro-apoptotic activity has been associated with the modulation of mitogen-activated protein kinase signaling and the B-cell lymphoma-2/caspase-3 signaling pathways [15]. Collectively, these findings highlight the considerable potential of shikonin as a therapeutic agent for the prevention and treatment of pathological scars. Despite its promising biological activities, the pharmaceutical application of shikonin remains challenging due to its poor physicochemical stability. The naphthoquinone structure of shikonin is highly susceptible to photodegradation and oxidative degradation upon exposure to environmental conditions. Previous research demonstrated that exposure to sunlight for one month resulted in the degradation of up to 96% of shikonin [16]. Therefore, the improvement of shikonin stability is essential for the successful development of effective pharmaceutical and topical formulations.

To overcome the photoinstability of shikonin, lipid-based delivery systems have been extensively investigated as a strategy to improve its stability and dermal delivery. Nanostructured lipid carriers (NLC) are a second-generation lipid nanoparticle system developed from solid lipid nanoparticles (SLN). NLC are composed of solid lipids, liquid lipids, and surfactants, allowing their biological behavior and efficacy to be tailored by adjusting their formulation [17]. The incorporation of liquid lipid into a solid–lipid matrix is generally expected to reduce solid lipid crystallinity and promote matrix imperfection in NLC systems. This imperfect crystal arrangement provides additional space within the matrix for the accommodation of active compounds, thereby enhancing drug loading capacity and preventing drug expulsion during storage, resulting in improved physical stability compared to the highly ordered crystalline structure of SLN [18,19]. Various methods have been employed for NLC preparation, including high-energy techniques, such as high-pressure homogenization, high-shear homogenization, and ultrasonication, as well as low-energy approaches, including emulsification and solvent evaporation methods [20,21]. Among these methods, high-energy techniques are widely used because of their scalability and compatibility with pharmaceutical manufacturing processes. NLC offer several advantages for topical drug delivery, including low toxicity, improved drug stability, reduced adverse effects of active compounds, enhanced skin penetration, and controlled release [22,23]. These characteristics make NLC particularly attractive for the delivery of shikonin to scar tissue and damaged skin.

Hydrogels are three-dimensional polymeric networks capable of absorbing and retaining large amounts of water while maintaining structural integrity [24]. In recent years, hydrogels have been extensively applied in biomedical and pharmaceutical applications. In particular, hydrogel-based dermal patches have attracted considerable interest as topical delivery systems. Patches provide controlled drug release over a prolonged period while maintaining extended contact of the drug or active compound with the skin surface [25]. Among the polymers commonly used in dermal formulations, hyaluronic acid (HA) and carboxymethyl cellulose (CMC) have demonstrated considerable potential. HA is a linear polysaccharide composed of repeating disaccharide units. The high molecular weight of HA enables its use as a controlled-release agent due to its high viscoelasticity and water-binding capacity [26]. CMC an anionic derivative of cellulose ether, is a nontoxic and biocompatible polymer capable of forming a film on the skin surface, thereby providing favorable mechanical properties for the use as a film-forming agent [27]. Recent studies have also demonstrated the successful incorporation of NLC into polymeric matrices to improve the stability and dermal delivery of bioactive compounds. For example, NLC have been incorporated into polymeric transdermal patches to enhance the dermal delivery and stability of capsaicin [28]. This approach highlights the potential of combining nanocarrier systems with hydrogel matrices to improve stability, membrane retention, and facilitate practical application on the skin. Therefore, this study aimed to develop and optimize shikonin-loaded NLC to improve the photostability of shikonin. The optimized NLC formulation was subsequently incorporated into a hydrogel matrix to fabricate a dermal patch, providing a potential alternative for topical scar treatment and self-care application.

## 2. Materials and Methods

### 2.1. Materials

Shikonin (≥98%) was acquired from ChemFaces Ind.Co., Ltd. (Wahun, China). Glyceryl distearate, sunflower oil, polysorbate 80 (Tween^®^ 80), sorbitan oleate (Span^®^ 80), propylene glycol (PG), polyethylene glycol 400 (PEG400), hyaluronic acid (HA; Large molecule, 2M Daltons), and carboxymethyl cellulose (CMC) were purchased from Chanjao Longevity (Bangkok, Thailand). Ethanol, methanol, acetonitrile (ACN) (HPLC grade), dimethyl sulfoxide (DMSO), formic acid (HPLC grade), tetrahydrofuran (THF) (HPLC grade), di-sodium hydrogen phosphate dihydrate (Na_2_HPO_4_·2H_2_O), sodium dihydrogen phosphate dihydrate (NaH_2_PO_4_·2H_2_O), sodium chloride (NaCl), and potassium chloride (KCl) were purchased from RCI Labscan (Bangkok, Thailand). Ortho-phosphoric acid was purchased from Merck Millipore (Berlin, Germany). Deionized (DI) water was used for the formulation and HPLC analysis.

### 2.2. Methods

#### 2.2.1. Identification of Shikonin

Shikonin was identified and quantitatively analyzed using a Shimadzu LC-2050C 3D high-performance liquid chromatography (HPLC) system (Shimadzu, Kyoto, Japan). Chromatographic analysis was performed under isocratic elution conditions adapted from the method reported by Albreht et al. (2009) [29]. Chromatographic separation was achieved using a KNAUER^®^ C-18 analytical column (Vertex Plus Column, 250 mm × 4.6 mm I.D., particle size 5 μm (Knauer Wissenschaftliche Geräte GmbH, Berlin, Germany). The mobile phase consisted of THF, ACN, DI water, and formic acid in a ratio of 30:20:50:0.5 (*v*/*v*). The injection volume was 10 μL. The flow rate was set at 1 mL/min, and the column temperature was maintained at 30 °C. The detection was performed at a wavelength of 518 nm. Each chromatographic run was carried out for 30 min. The shikonin standard solution was prepared at concentrations of 0.78 to 400 ppm.

#### 2.2.2. Solubility Study of Shikonin

The solubility of shikonin in various solvents was evaluated using a modified miniaturized shake-flask method adapted from Veseli et al. [30]. Briefly, shikonin (0.1 mg) was weighed using an analytical balance (OHAUS PX224 PIONEER, Los Angeles, CA, USA) and transferred into a microcentrifuge tube containing 1 mL of each solvent, including DI water, ethanol, DMSO, PG, and PEG400. Each sample was mixed for 1 min. The dissolution of shikonin was visually observed to ensure complete dissolution. Subsequently, 0.1 mg shikonin was added incrementally until a total amount of 0.5 mg was reached. After each addition, the samples were then sonicated for 10 min. After sonication, the samples were wrapped in aluminum foil and refrigerated at 4 °C. They were left overnight for 24, 48, and 72 h. Solubility was visually assessed for precipitation or undissolved particles. Quantitative determination of dissolved shikonin was subsequently performed using the HPLC method described in Section 2.2.1.

#### 2.2.3. Development of NLC Formulations

##### Development of Blank NLC with Varying Lipid Contents

Blank NLC were fabricated using a high-energy emulsification method combining high-shear homogenization and ultrasonication [31,32]. The lipid phase consisted of solid lipid and liquid lipid at a fixed ratio of 3:2. The total lipid concentration was varied at 5, 10, 15, and 20% *w*/*w*. The aqueous phase composed of DI water and polysorbate 80 was heated to 75 °C. The lipid phase composed of glyceryl distearate (solid lipid), sunflower oil (liquid lipid), and sorbitan oleate was heated to 70 °C until completely melted. The heated aqueous phase was gradually added to the molten lipid phase under continuous stirring to form a coarse emulsion. These compositions were adapted from Jandang et al. (2024) [22], as shown in Table 1. The resulting coarse emulsion was homogenized using a high-shear homogenizer (IKA^®^ T25 digital Ultra-Turrax, Staufen, Germany) at 10,000 rpm for 10 min. Subsequently, the particle size reduction was achieved using a sonicator probe (Sonics and Materials, Newtown, CT, USA) at 60% amplitude under pulse mode (3 s on /3 s off) for 15 min. The prepared NLC formulations were initially evaluated for physical stability based on the presence or absence of phase separation after 24 h of storage. The effects of lipid composition on particle size and polydispersity index (PDI) were systematically investigated to identify the optimal formulation.

##### Development of Blank NLC with Varying Mixed Surfactant Ratios

The appropriate blank NLC obtained from the lipid screening study was further optimized by varying the ratio of mixed surfactants (total concentration of 20% *w*/*w*), as shown in Table 2. All formulations were prepared according to the NLC preparation procedure described in previous section. The resulting NLCs were characterized in terms of particle size, PDI, and physical stability to identify the optimal surfactant composition.

##### Characterization of NLC Formulations

The particle size, PDI, and zeta potential of NLC formulations were measured using a Zetasizer^®^ Nano Zs (Malvern, Worcestershire, UK). The sample was diluted at a ratio of 1:100 with DI water before the analysis, at 25 ± 0.5 °C using a detection angle of 173° [33].

##### Stability Study of Blank NLC Formulations

The physical stability of blank NLC formulations was evaluated after storage at room temperature (30 °C) for one month. Physical appearances were visually assessed for signs of phase separation, creaming, sedimentation, or aggregation. In addition, particle size, PDI, and zeta potential were measured before and after the stability test.

##### Development of Shikonin-Loaded NLC

The NLC formulation with small particle size, narrow PDI, and good stability was selected for subsequent loading of shikonin. The shikonin-loaded NLC consisted of 3% glyceryl distearate, 2% sunflower oil, 6.67% sorbitan oleate, 13.33% polysorbate 80, 5% PG, and DI water q.s. to 100% (*w*/*w*). A PG concentration of 5% was selected as the optimal concentration based on preliminary experiments using blank NLC with varying concentrations of PG (5%, 10%, and 15%). This concentration provided the smallest particle size while maintaining an acceptable PDI. Therefore, this concentration was subsequently used as a solubilizing agent for shikonin. Shikonin was dissolved in PG (0.06% *w*/*w*) by vortexing for 1 min, followed by sonication for 5 min at room temperature. The solution was incorporated into the melted lipid phase before mixing with the aqueous phase, giving a shikonin concentration of 0.003% *w*/*w* in the 100 g batch.

##### Determination of Entrapment Efficiency of Shikonin-Loaded NLC

The percentage of entrapment efficiency (%EE) of shikonin-loaded NLC was determined using an indirect method. The sample was divided into two parts. First, 500 µL of shikonin-loaded NLC was mixed with 500 µL of methanol in a microcentrifuge tube (total compound added). The mixture was then centrifuged (Thermo Fisher Scientific, Sorvall ST16R, Merck, Darmstadt, Germany) at 10,000 rpm (6755× *g*) for 30 min at room temperature. The supernatant was collected for HPLC analysis. In the second part, 500 µL of shikonin-loaded NLC was added into an ultrafiltration tube (Amicon^®^ Ultra-0.5 mL, 50 kDa MWCO, Merck, Darmstadt, Germany) with a pore size of 10 to 100 nm [32], and then centrifuged at 10,000 rpm for 30 min at room temperature. The unloaded shikonin solution was dropped into the bottom of the ultrafiltration tube (non-entrapped compound). After that, the amount of shikonin in the total compound added and non-entrapped compound was analyzed using HPLC (Shimadzu, Kyoto, Japan). The %EE of shikonin in NLC was calculated by the following equation: [22,34].

(1) $$\%\mathrm{EE}=\;\frac{\left(\mathrm{Total}\;\mathrm{compound}\;\mathrm{added}\;-\;\mathrm{Non}-\mathrm{entrapped}\;\mathrm{compound}\right)\;}{\mathrm{Total}\;\mathrm{compound}\;\mathrm{added}}\;\times 100$$

##### Stability Study of Shikonin-Loaded NLC

The physical stability of each shikonin-loaded NLC was assessed under accelerated storage conditions. The formulations were subjected to six heating–cooling cycles. During each cycle, the formulations were stored at 45 °C for 24 h and subsequently stored at 4 °C for an additional 24 h. In addition, the formulations were stored at 30 °C, 4 °C, and 45 °C for 30 days. The physicochemical properties of the formulations were evaluated at predetermined time points (Day 0 and Day 30). The evaluated parameters included physical appearance, color, particle size, PDI, and zeta potential.

##### Morphology of Shikonin-Loaded NLC

The morphology of blank NLC and shikonin-loaded NLC was observed using transmission electron microscopy (TEM; a JEOL JEM-2100 Electron Microscope, Tokyo, Japan). The sample was diluted with DI water in a ratio of 1:100 before being dropped onto a carbon-coated copper grid (Carbon film 300 mesh, Sacramento, CA, USA). The grids were allowed to air-dried for 15 min. The samples were negatively stained with 3% (*w*/*v*) phosphotungstic acid prepared in DI water. Excess staining solution was removed using 0.22 µm Millipore^®^ filter paper, and the grid was examined by TEM at 100 kV and 30,000× magnification [34]. Then, the particle size of blank NLC and shikonin-loaded NLC were determined from the TEM image using ImageJ software (version 1.54g, Wayne Rasband and contributors, National Institutes of Health, Bethesda, MD, USA). Briefly, the image scale was established according to the scale bar embedded in each TEM image using the set scale instrument. Nanoparticle diameters were then obtained by measuring a straight line across the widest distance of an individual nanoparticle.

#### 2.2.4. Photostability Test of Shikonin-Loaded NLC

The photostability test was performed as a comparative photostability screening experiment [33,35,36]. Shikonin in PG solution and shikonin-loaded NLC were prepared and exposed to near-UV radiation (200–400 nm) and white light in a UV cabinet, providing a total illumination intensity of 72,000–84,000 lux. Briefly, 2 mL of each sample was placed onto a glass plate and positioned at a fixed distance of 15 cm from the light source within a light-tight wooden cabinet [37]. Samples were collected at predetermined time intervals (0, 2, 4, 6, and 8 h) for quantitative analysis of the remaining shikonin content. To mitigate potential concentration changes resulting from solvent evaporation during the light exposure period, the shikonin-loaded NLC were adjusted to their initial volume using DI water prior to analysis. For the analysis of shikonin-loaded NLC, the collected samples were mixed with methanol at a ratio of 1:1 in an ultracentrifuge tube. The mixtures were vortexed, centrifuged, and collected the supernatant for HPLC analysis.

#### 2.2.5. In Vitro Release Study of Shikonin-Loaded NLC

The release study of shikonin in PG solution and shikonin-loaded NLC were assessed using a dialysis bag diffusion method adapted from Arunprasert et al. (2022) [28]. Samples at a concentration of 0.1 mg/mL (1 mL) were placed in a dialysis bag (CelluSep^®^ T2, Seguin, TX, USA; molecular weight cut-off 6–8 kDa) with a surface contact area of 2 cm × 2 cm. The dialysis bags were pre-conditioned by immersion in the release medium for 24 h prior to the experiment to ensure that the dialysis membrane itself did not act as the rate-limiting step. The study was conducted at 37 °C ± 2 °C using a release medium consisting of phosphate-buffered saline (PBS, pH 7.4) and ethanol (70:30, *v*/*v*; 10 mL) in a chamber to maintain sink conditions. Throughout the experiment, the medium was continuously agitated at 300 rpm to ensure uniformity. During the study, 1 mL of the release medium was periodically withdrawn from the chamber using an autopipette at predetermined time intervals (0.5, 1, 2, 4, 6, 8, and 12 h). To maintain a constant volume, an equal volume (1 mL) of fresh medium was immediately replenished using the same precision pipette. The amount of shikonin released into the medium was quantified using the HPLC method described previously. The cumulative percentage of shikonin released was subsequently calculated and used to evaluate the release behavior of the formulations.

#### 2.2.6. Development of Hydrogel-Based Dermal Patch

##### Preparation of Hydrogel-Based Dermal Patch

The compositions of the hydrogel-based dermal patches are presented in Table 3. HA and CMC were selected as the polymeric matrix for patch preparation. Hydrogel-based dermal patches were prepared by a solvent casting technique. Each polymer was dissolved in DI water at a total concentration of 1% *w*/*w*. The polymer solution was continuously stirred at 300 rpm for 1 h using a magnetic stirrer (IKA-Werke GmbH & Co. KG, Staufen im Breisgau, Germany). Then, ethanol and PG were added to the polymer solution. After that, the polymer solution was continuously stirred for uniform mixing and distribution until a hydrogel-based was achieved. Then, to remove the entrapped air bubbles, the samples were placed in a sonicator bath for 15 min. The formulation was poured into a silicone mold (Ice House, Sa Kaeo, Thailand) with a surface area of 57.34 cm^2^. Then, the formulations were dried in a hot air oven at 45 °C for 8 h. After the drying process, the hydrogel-based dermal patches were peeled off from the silicon molds. To control the environmental conditions, each sample was individually sealed in aluminum foil sachet and stored at room temperature for 24 h before evaluation.

##### Characterization of Hydrogel-Based Dermal Patch

The hydrogel-based dermal patches were characterized to evaluate their physicochemical and mechanical properties. All experiments were conducted under controlled laboratory conditions at 25 °C and 40–55% relative humidity. All investigations were done in triplicate.

##### Physical Appearances

The physical appearances of hydrogel-based dermal patches were investigated by visual inspection. Parameters including color, transparency, surface smoothness, homogeneity, and the presence of visible defects were assessed.

##### pH Measurement

The pH of the formulations before casting film was measured using a pH meter (Starter 2100, OHAUS, Parsippany, NJ, USA).

##### Thickness

The thickness of the hydrogel-based dermal patches was measured by a digital vernier caliper (INSIZE 1108-200, Bangkok, Thailand). The patch size 6 × 9 (cm) was evaluated at three standardized positions, including the center and two opposite peripheral points. The mean thickness of each patch was calculated from the average of the three measurements [38].

##### Adhesive Capability

The adhesive capability of the hydrogel-based dermal patches was evaluated using a texture analyzer (Stable Micro systems, TA-XT plus, Godalming, UK). The adhesion strength was measured in triplicate using a P/20P cylindrical probe (20 mm diameter) at 25 °C. The contact time between the probe and the sample surface was set at 1 s. The maximum force required to detach the probe from the patch was recorded as the adhesion strength (N) [29]. The adhesion data was used to assess the ability of the hydrogel-based dermal patches to maintain adequate contact with the skin surface.

##### Tensile Strength and Elongation at Break

Tensile strength (MPa) and elongation at break (%) of the hydrogel-based dermal patches were evaluated using a texture analyzer following the ASTM standard method (D882-02, 2002) [39] with slight modifications. The patches were cut into rectangular strips, 2 cm × 9 cm. The initial grip separation and crosshead speed were set at 30 mm and 1 mm/s, respectively [40]. Tensile strength and percent elongation at break were reported as the mean values obtained from three independent measurements. The tensile strength and elongation at break were calculated using the following equations.

(2) $$\mathrm{Tensile}\;\mathrm{strength}=\frac{\mathrm{Breaking}\;\mathrm{force}\;(N)}{\mathrm{Cross}-\mathrm{Sec}\mathrm{tional}\;\mathrm{area}\;\mathrm{of}\;\mathrm{sample}\;({\mathrm{mm}}^{2})}$$

(3) $$\%\mathrm{Elongation}\;\mathrm{at}\;\mathrm{break}=\frac{\mathrm{Increase}\;\mathrm{in}\;\mathrm{length}\;\mathrm{at}\;\mathrm{breaking}\;\mathrm{point}\;(\mathrm{mm})}{\mathrm{Original}\;\mathrm{length}\;(\mathrm{mm})}\;\times \;100$$

#### 2.2.7. Development of Hydrogel-Based Dermal Patch Containing Shikonin-Loaded NLC

##### Preparation of Hydrogel-Based Dermal Patch Containing Shikonin-Loaded NLC

To evaluate the compatibility between the blank NLC formulation and the hydrogel, the blank NLC formulation was incorporated into the hydrogel at various ratios, as summarized in Table 4. Then, the mixture was evaluated by visual inspection for signs of phase separation, aggregation, precipitation, or other physical instabilities after 24 h. Subsequently, the particle size, PDI, and zeta potential of the NLC-based hydrogel were measured using Zetasizer^®^(Malvern, Worcestershire, UK). After that, the NLC-based hydrogel was poured into a glass plate and dried using a hot-air oven (Memmert, Büchenbach, Germany). The resulting hydrogel-based dermal patches were evaluated for their characteristics including appearance, color, and texture. Based on the compatibility and physical characterization results, the optimal blank NLC-to-hydrogel ratio was selected for the preparation of hydrogel-based dermal patches containing shikonin-loaded NLC.

##### Characterization of Hydrogel-Based Dermal Patch Containing Shikonin-Loaded NLC

Hydrogel-based dermal patches containing shikonin-loaded NLC were evaluated for physical appearance, pH, color, weight, thickness, and adhesive property.

##### Stability of Hydrogel-Based Dermal Patch Containing Shikonin-Loaded NLC

The stability study of the hydrogel-based dermal patch containing shikonin-loaded NLC was investigated before and after storage at 30 °C for 30 days. In addition, physical appearance, pH, color, weight, thickness, and adhesive property were measured before and after the stability test.

#### 2.2.8. Membrane Retention and Permeation Study of Hydrogel-Based Dermal Patch Containing Shikonin-Loaded NLC

The in vitro membrane retention and permeation study was evaluated using Franz diffusion cells (Logan Instruments, Somerset, NJ, USA) with some modifications [41]. This study was to investigate the permeation and retention of shikonin released from a hydrogel dermal patch containing shikonin-loaded NLC through the Strat-M^®^ membrane (Strat-M^®^ EMD Millipore, Burlington, MA, USA). Both the shikonin solution and the hydrogel-based dermal patch containing shikonin-loaded NLC contained an equivalent shikonin concentration of 0.03 mg. One milliliter of shikonin solution containing 0.03 mg/mL was applied to the donor chamber. A dried hydrogel-based dermal patch containing shikonin-loaded NLC and providing an equivalent shikonin dose of 0.03 mg/g was fully hydrated with water before being placed on the membrane. The effective permeation area was 3.14 cm^2^. The receptor chamber was filled with 13 mL of PBS (pH 7.4) mixed with ethanol at a volume ratio of 70:30 (*v*/*v*) and maintained at 37 ± 2 °C under continuous magnetic stirring at 300 rpm. A 1 mL aliquot from the receptor chamber was withdrawn at 0.5, 1, 2, 4, 6, 8, 12, and 24 h, and fresh medium was immediately added with an equal volume to maintain the initial volume. The quantity of shikonin permeated into the receptor chamber at different periods of time points was analyzed using HPLC. After 24 h, the membrane was gently rinsed with PBS to remove residual formulation from its surface, and cut into small pieces. The remained shikonin in membrane was extracted with methanol using ultrasonic bath for 15 min. The concentration of shikonin in the membrane was analyzed using HPLC.

#### 2.2.9. Statistical Analysis

All experiments were performed in triplicate, and the results are presented as mean ± SD. Statistical analyses were conducted using SPSS software (version 17.0). Comparisons between two groups were performed using Student’s *t*-test, whereas comparisons among multiple groups were analyzed using one-way analysis of variance (ANOVA) with Tukey post hoc test. A two-way analysis of variance (ANOVA), followed by Tukey post hoc test, was performed to evaluate differences among groups. Differences were considered statistically significant at *p* < 0.05.

## 3. Results and Discussion

### 3.1. HPLC Analysis of Shikonin

**Figure 2:**
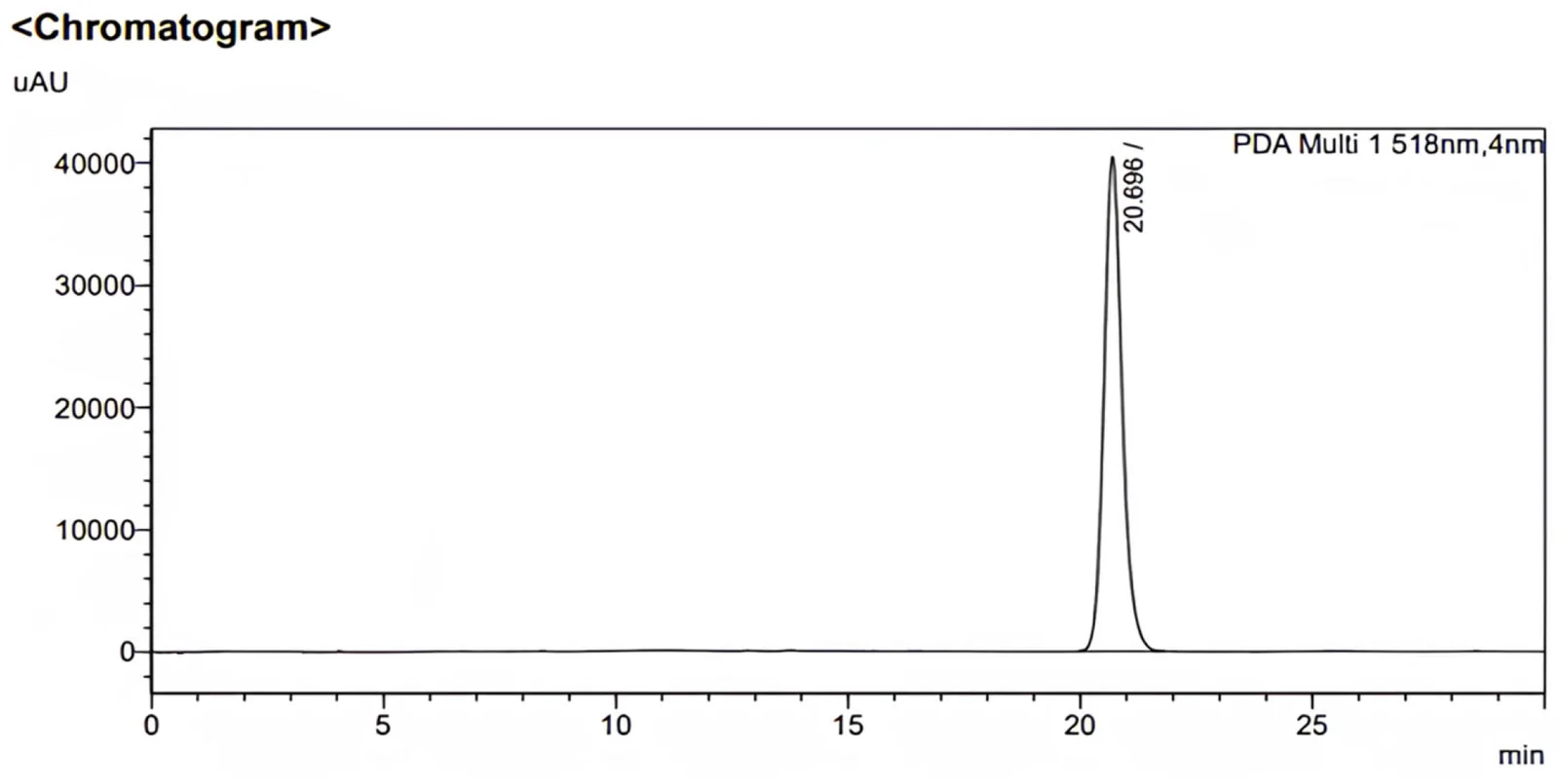
HPLC chromatogram of shikonin.

**Figure 3:**
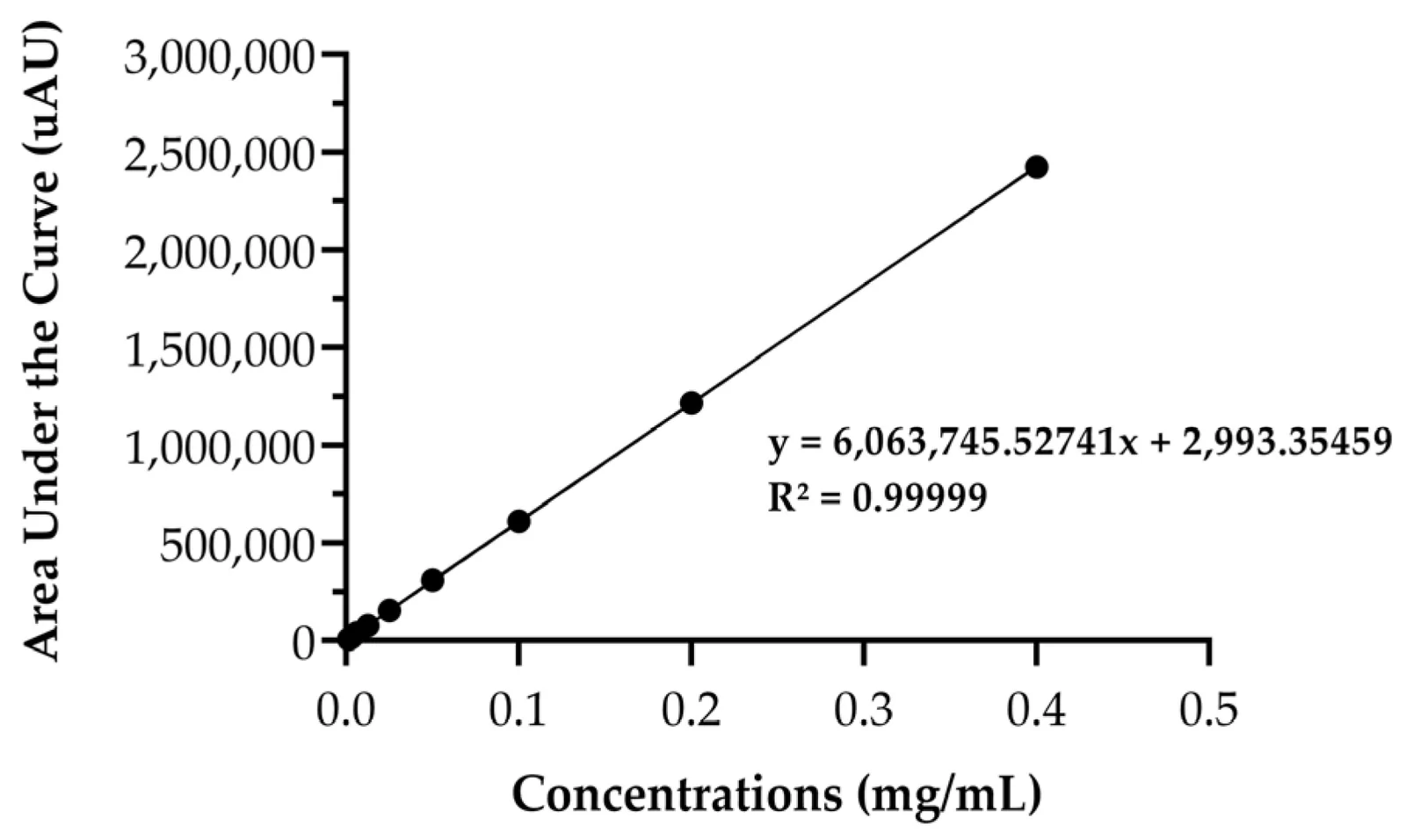
Calibration curve of shikonin.

Shikonin was successfully detected at a wavelength of 518 nm and exhibited a well-defined peak with a retention time of approximately 20 min within the 30 min as shown in Figure 2. The chromatographic condition enabled clear identification and quantification of shikonin. The calibration curve demonstrated excellent linearity over the tested concentration range, with a correlation coefficient (R^2^) of 0.9999 (Figure 3), indicating the suitability of the method for quantitative analysis.

### 3.2. Solubility of Shikonin

**Figure 4:**
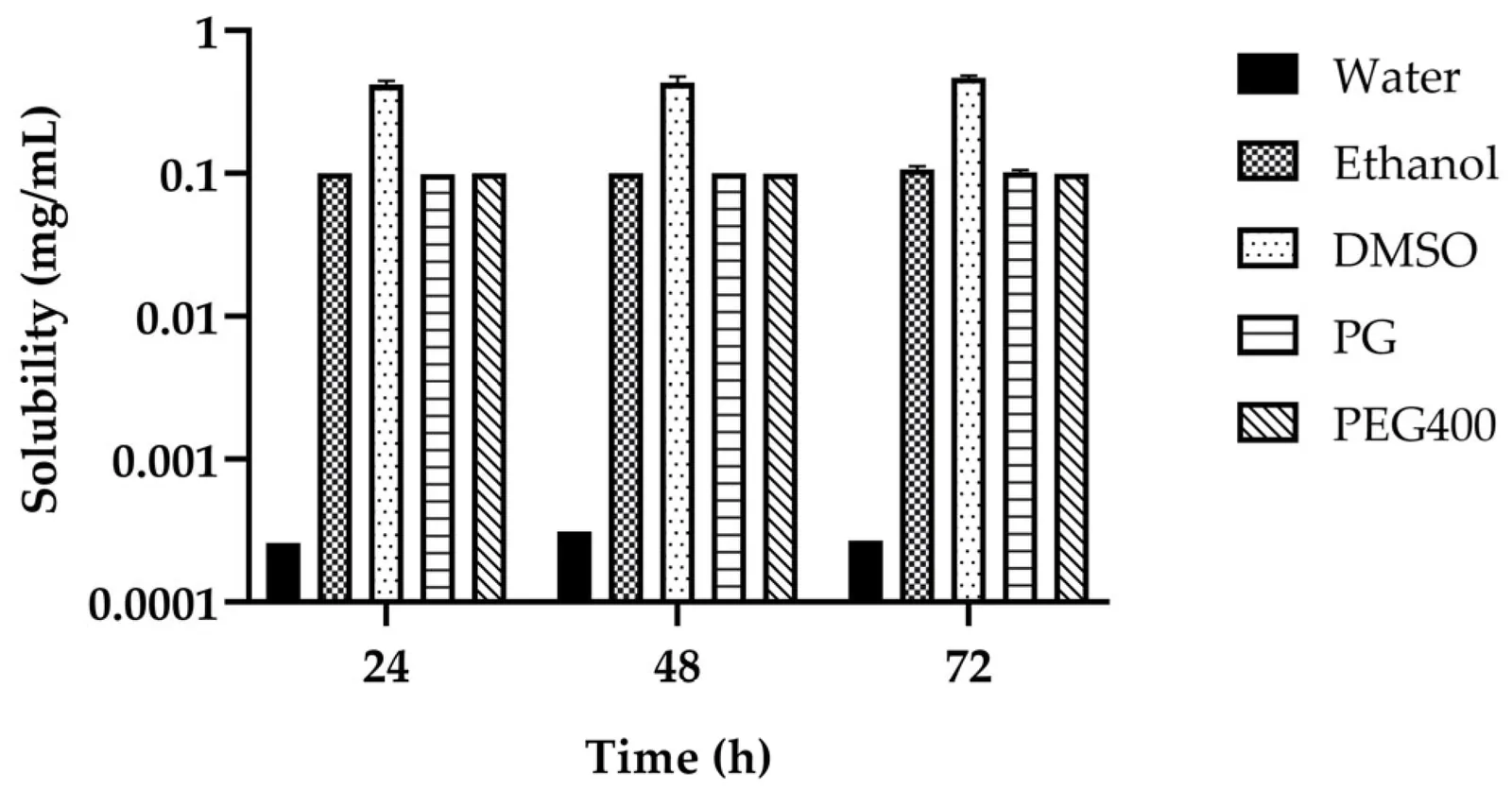
Solubility of shikonin in water, ethanol, DMSO, PG, and PEG400 after 24, 48, and 72 h. Data are expressed as mean ± SD (*n* = 3).

The solubility of shikonin was evaluated in various solvents. The results are presented in Figure 4. Shikonin exhibited good solubility in DMSO with no visible precipitation observed immediately after the solvent was added and throughout the storage period. Ethanol, PG, and PEG400 exhibited initial precipitation. However, the precipitates were completely dissolved upon sonication, resulting in no visible precipitation. In contrast, DI water showed the poorest solubility, with persistent precipitation observed at 24, 48, and 72 h. The poor solubility of shikonin in DI water is consistent with hydrophobic structure of shikonin and its limited affinity toward highly polar aqueous media. In contrast, DMSO, ethanol, PG, and PEG400 possess intermediate-to-high solvent polarity and hydrogen-bonding capability, which facilitate the solubilization of shikonin. Although DMSO provided the highest solubilizing capacity, it was not selected for further formulation development because of concerns regarding its toxicity. Previous clinical dermatological study has reported that DMSO may cause itching, skin irritation, tingling or burning, and barrier disruption [42]. In addition, DMSO acts as a potent penetration enhancer by disrupting the lipid organization of the stratum corneum. This effect is attributed to its interaction with the intercellular lipid matrix, where it disrupts lipid packing and alters intracellular keratin domains [43]. Despite demonstrating the second-highest solubility and its widespread use in formulations, ethanol was not selected for further development due to concerns regarding its potential to compromise epidermal barrier integrity, increase stratum corneum permeability, and elevate transepidermal water loss, thereby increasing the risk of skin irritation [44]. Among the investigated solvents, PG was selected as an appropriate solvent for shikonin incorporation in the NLC formulation. Since the developed formulation was intended for dermal application, solvent selection was based not only on the solubilization capacity of shikonin but also on considerations related to skin compatibility, safety, and formulation suitability. PG possessed a favorable safety profile, low irritation potential, and widespread use in topical pharmaceutical formulations [45]. In addition, PG has been reported to enhance the long-term physical stability of NLC formulations [46].

### 3.3. Development of NLC Formulation

#### 3.3.1. Blank NLC with Varying Lipid Contents

**Figure 5:**
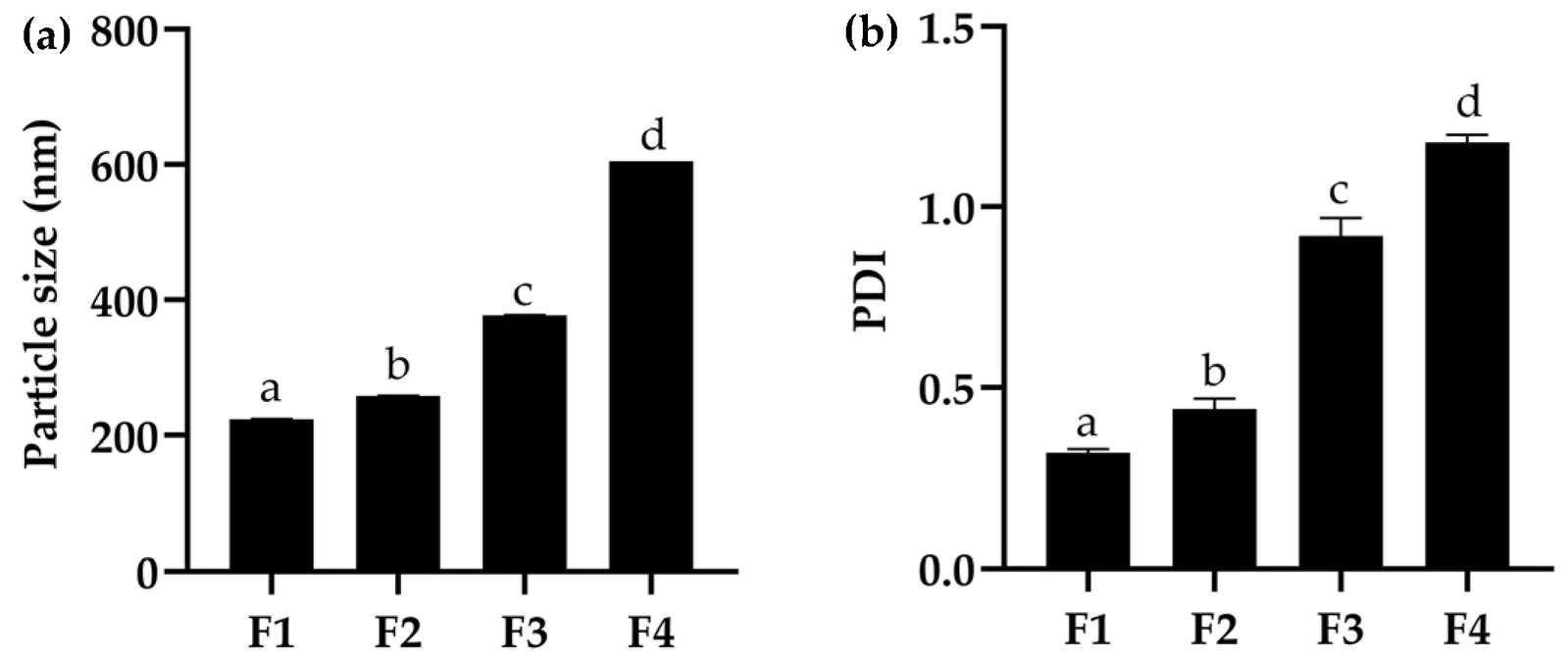
Particle size (**a**) and PDI (**b**) of formula 1 (F1), formula 2 (F2), formula 3 (F3), and formula 4 (F4). Data are expressed as mean ± SD (*n* = 3). Different letters (a–d) indicate statistically significant differences among samples (*p* < 0.05), as determined by One-way ANOVA followed by Tukey post hoc test.

The preliminary development of blank NLC formulations (F1–F4) was conducted to investigate the effect of lipid content (5–20% *w*/*w*) on the physicochemical properties of the formulations. Glyceryl distearate was used as a solid lipid and sunflower oil was used as a liquid lipid. Sorbitan oleate and polysorbate 80 were used as emulsifiers at fixed total concentration of 20% (*w*/*w*). The particle size and PDI of all formulations are presented in Figure 5. All formulations (F1–F4) showed particle sizes larger than 200 nm in the range of 223 to 605 nm, with PDI values ranging from 0.3 to 1.1. The PDI of nanomaterials can be divided into three categories as follows: values between 0.1 and 0.3 indicate well-dispersed systems, values between 0.3 and 0.5 indicate moderately dispersed systems, and values between 0.5 and 1.0 suggest polydispersity which might occur aggregation [47]. Formula F1 contained a total lipid content of 5%, exhibited the smallest particle size of 223.85 ± 0.32 and a PDI value of 0.32 ± 0.01. Compared to the other formulations (F2–F4), it clearly demonstrated that increasing the lipid content to 10, 15, and 20% resulted in a significant increase in particle size. This phenomenon may be attributed to the increased viscosity of the lipid phase at higher lipid concentrations, which can reduce the efficiency of particle size reduction during homogenization and ultrasonication. In addition, increasing the lipid content without a corresponding increase in surfactant concentration may result in insufficient surface coverage of newly generated lipid particles, thereby promoting particle growth and aggregation [48]. Among the investigated formulations, formula F1 exhibited the most favorable physicochemical characteristics. Therefore, it was selected for further study of the development of NLC formulation.

#### 3.3.2. Blank NLC with Varying Mixed Surfactant Ratios

**Figure 6:**
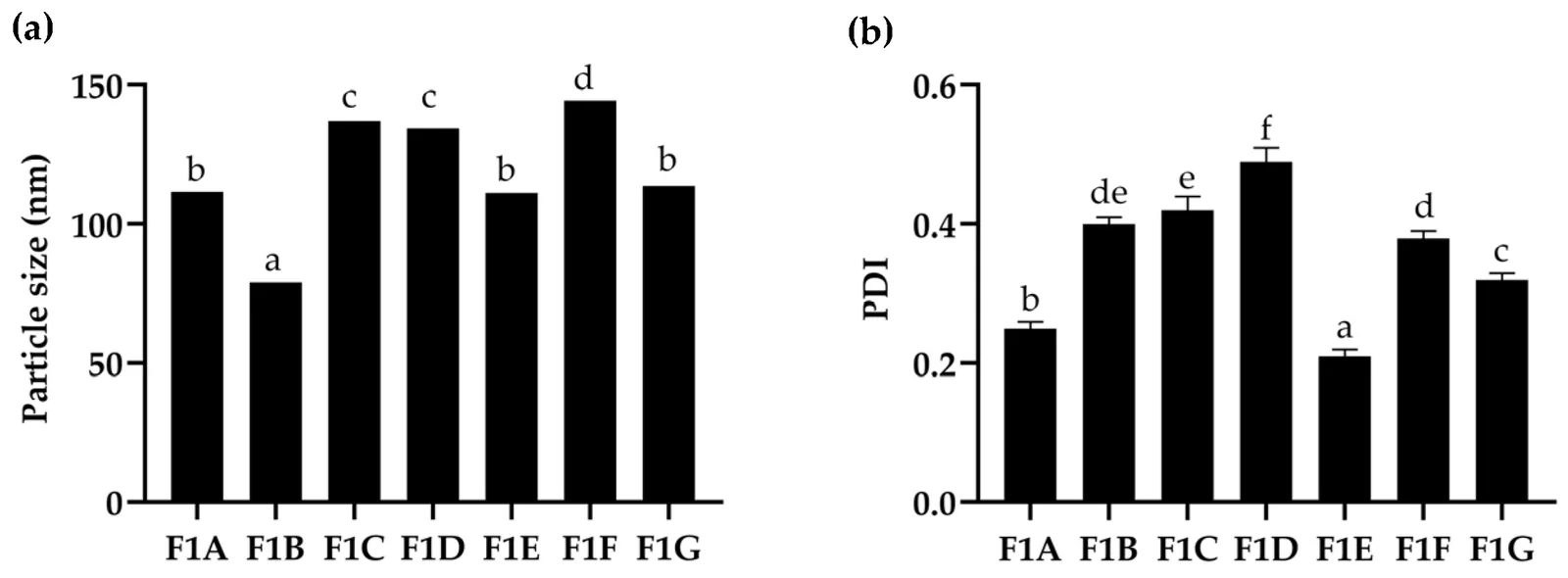
Effect of varying mixed surfactant ratios on particle size (**a**) and PDI (**b**) of NLC formulations. Data are expressed as mean ± SD (*n* = 3). Different letters (a–f) indicate statistically significant differences among samples (*p* < 0.05), as determined by One-way ANOVA followed by Tukey post hoc test.

The effects of varying mixed surfactant ratios on the physicochemical properties of blank NLC formulations are presented in Figure 6. All formulations exhibited a reduced particle size following adjustments in the surfactant ratios, with particle size in the range of 79 to 144 nm. Although the total surfactant concentration was fixed at 20% *w*/*w*, altering the ratio between sorbitan oleate and polysorbate 80 significantly influenced particle size and size distribution. The optimization of mixed surfactant ratios was performed because surfactant composition critically affected the formation, physicochemical properties, and stability of NLC. An appropriate combination of surfactants can effectively reduce interfacial tension between the lipid and aqueous phases, facilitate nanoparticle formation, reduce particle aggregation, and enhance steric and electrostatic stabilization. These effects contribute to a narrower particle size distribution and improved colloidal stability of the formulation [49]. The combination of sorbitan oleate and polysorbate 80 reduces the interfacial tension between the lipid droplets and aqueous phase, thereby improving emulsification efficiency and facilitating the formation of smaller nanoparticles [50,51]. Formulation F1B exhibited the smallest particle size of 79 nm, whereas F1E showed the lowest PDI value of 0.21. Although F1A, F1E, and F1G showed comparable particle sizes and narrow PDI values, statistical analysis revealed that F1E showed a significantly lower PDI than the other formulations. The PDI value of F1B showed higher (0.40) than F1E that indicated a broader particle size distribution and lower homogeneity. This phenomenon may be explained by excessive particle disruption under high shear homogenization emulsification conditions, which leads to a wider particle size distribution and recoalescence [52]. Conversely, F1E exhibited a slightly larger particle size but maintained a PDI value below 0.3, indicating a more uniform particle distribution and improved colloidal stability [53]. In addition, F1C, F1D, and F1F exhibited larger particle size and PDI value (>0.3), which was inconsistent with the desired criteria for formulation selection. PDI is a critical parameter reflecting formulation homogeneity and long-term physical stability [49]. Therefore, F1E was selected for further development.

#### 3.3.3. Characterization and Stability of Blank NLC and Shikonin-Loaded NLC

**Figure 7:**
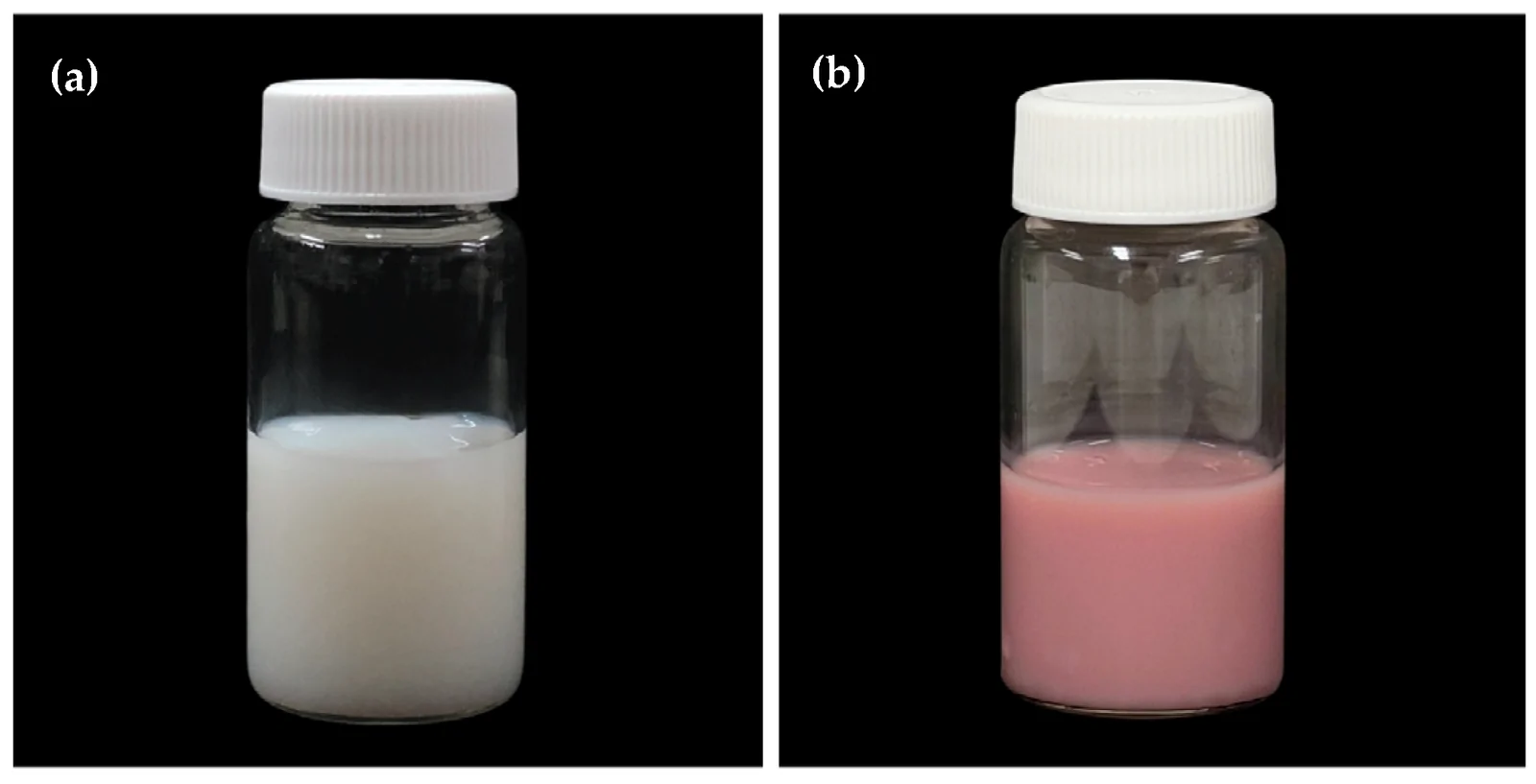
Physical appearance of blank NLC (**a**) and shikonin-loaded NLC (**b**).

The physical appearance of blank NLC and shikonin-loaded NLC are represented in Figure 7. Both formulations were homogeneous and showed no visible aggregation. The blank NLC exhibited a milky appearance, whereas the shikonin-loaded NLC exhibited a pink appearance. As can be seen, particle size of blank NLC decreased from 111 nm (F1E) to 78 nm. This reduction may be attributed to the incorporation of PG into the formulation. This observation is consistent with a previous study, which demonstrated that the inclusion of PG in an NLC formulation contributes to a smaller particle size [46]. Blank NLC formulation was evaluated for its stability at room temperature (30 °C) over a one-month storage period. The particle size, PDI, and zeta potential of the blank NLC before and after the stability test are presented in Table 5. The particle size decreased significantly from 78.41 ± 0.01 nm to 73.73 ± 0.01 nm (*p* = 0.003), while the PDI remained statistically unchanged (*p* = 0.943). However, the values remained within an acceptable range. The zeta potential of the blank NLC was significantly changed after the stability test. The blank NLC showed a negative surface charge surrounding each nanoparticle, providing a good repulsive force between the particles to reduce aggregation. The negative surface charge obtained from the carboxyl functional group of glyceryl distearate and sunflower oil generates a negative zeta potential [22].

The particle size, PDI, and zeta potential of shikonin-loaded NLC before and after stability test are presented in Table 6. The initial particle size was 104.94 ± 0.01 nm. The particle size ranged from 94.95 to 111.21 nm after storage for 1 month under various conditions, indicating that the formulation remained within the acceptable nanoscale range without extensive particle aggregation. The particle size observed at room temperature and 45 °C resulted in an increase in particle size, which may be attributed to Ostwald ripening at an elevated temperature [54], suggesting the formulation should be stored at 4 °C. Meanwhile, the reduction in particle size observed under certain conditions may be associated with lipid matrix rearrangement within the NLC system. In addition, the heating–cooling condition resulted in a reduction in particle size, which may be attributed to lipid matrix reorganization during the heating and cooling cycle. All formulations maintained PDI values below 0.3, indicating narrow particle size distribution and good homogeneity of the NLC system. In addition, the zeta potential values ranged from −23.51 to −33.80 mV, suggesting sufficient electrostatic repulsion and good colloidal stability. The chemical stability of shikonin in the NLC formulation was evaluated by determining drug recovery (%) after 30 days of storage under different conditions. The 4 °C condition showed the highest drug recovery (93.66 ± 3.26%), followed by RT (87.46 ± 9.61%) and 45 °C (85.14 ± 1.14%), while H/C cycling showed the lowest drug recovery (77.71 ± 4.99%). These results suggest that repeated thermal cycling imposes greater degradative stress on shikonin than constant elevated temperature, likely due to repeated phase transitions of the lipid matrix that disrupt the solid–lipid network and promote drug leakage or degradation. This trend was consistent with the more pronounced changes in particle size observed under H/C conditions.

#### 3.3.4. Entrapment Efficiency of Shikonin-Loaded NLC

The entrapment efficiency (%EE) of shikonin-loaded NLC was found to be 93.77 ± 2.05%, indicating excellent encapsulation of shikonin within the lipid nanocarrier system. The total-drug extraction procedure using a 1:1 mixture of NLC and methanol showed a percent recovery of 82.18 ± 3.37%. The high %EE can be attributed to the components and structural characteristics of the NLC formulation. Glyceryl distearate acted as the solid lipid, providing the primary structural matrix of the formulation. Sunflower oil functioned as the liquid lipid, which could reduce the crystallinity of the lipid matrix and consequently lower the melting point of the lipid system. This imperfect lipid arrangement generated additional space within the matrix, facilitating the incorporation and retention of shikonin and consequently enhancing entrapment efficiency. In addition, shikonin is a lipophilic compound with a reported logarithm of the partition coefficient (log P) value between 2.44 and 4.35 [55,56], indicating high affinity for the lipid phase. Therefore, shikonin could be effectively incorporated and retained within the oil phase of the formulation. Furthermore, PG was used as solvent for shikonin. PG exhibits good compatibility with both the aqueous and lipid phases due to its amphiphilic nature, containing hydrophilic hydroxyl groups and a lipophilic hydrocarbon chain. These properties facilitated the incorporation and stabilization of shikonin within the NLC system, thereby contributing to the overall stability and encapsulation efficiency of the formulation.

#### 3.3.5. Morphology of Blank NLC and Shikonin-Loaded NLC

**Figure 8:**
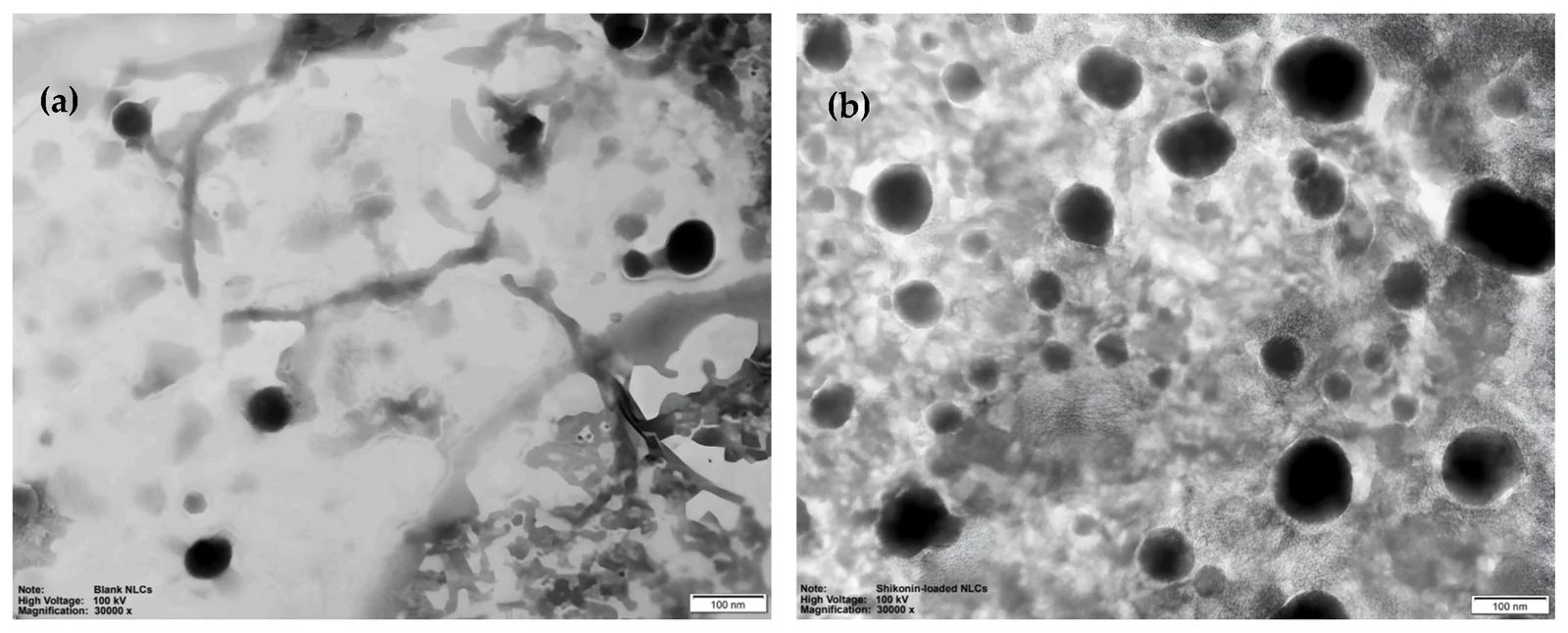
Images of blank NLC (**a**) and shikonin-loaded NLC (**b**) obtained from transmission electron microscope (TEM).

The TEM images of the blank NLC and shikonin-loaded NLC are presented in Figure 8. Both formulations were spherical with smooth surfaces, indicating the successful preparation of NLC using a high-shear homogenization and probe sonication method. Particle size analysis from the TEM images using ImageJ showed average sizes of 75.11 ± 6.70 nm and 102.26 ± 2.18 nm for the blank NLC and shikonin-loaded NLC, respectively. These values were slightly smaller than the corresponding hydrodynamic diameters measured by DLS (Zetasizer, Malvern, Worcestershire, UK), which were 78.41 ± 0.39 nm for the blank NLC and 104.94 ± 0.01 nm for the shikonin-loaded NLC. Nevertheless, the results showed good agreement between the two techniques. The observed differences may be attributed to their distinct measurement principles: DLS measures the hydrodynamic diameter of particles in a hydrated state, which includes the surfactant and solvation layers, whereas TEM measures the dry-state core diameter of individual particles under high vacuum [57]. These results are consistent with findings from previous study of NLC nanoparticles, confirming that the observed size difference is consistent with the established differences between hydrated and dry-state measurements [58]. Furthermore, the increase in particle size following shikonin encapsulation, observed in both techniques, may be attributed to the drug being accommodated within the lipid matrix, leading to structural expansion.

### 3.4. Photostability of Shikonin Solution and Shikonin-Loaded NLC

**Figure 9:**
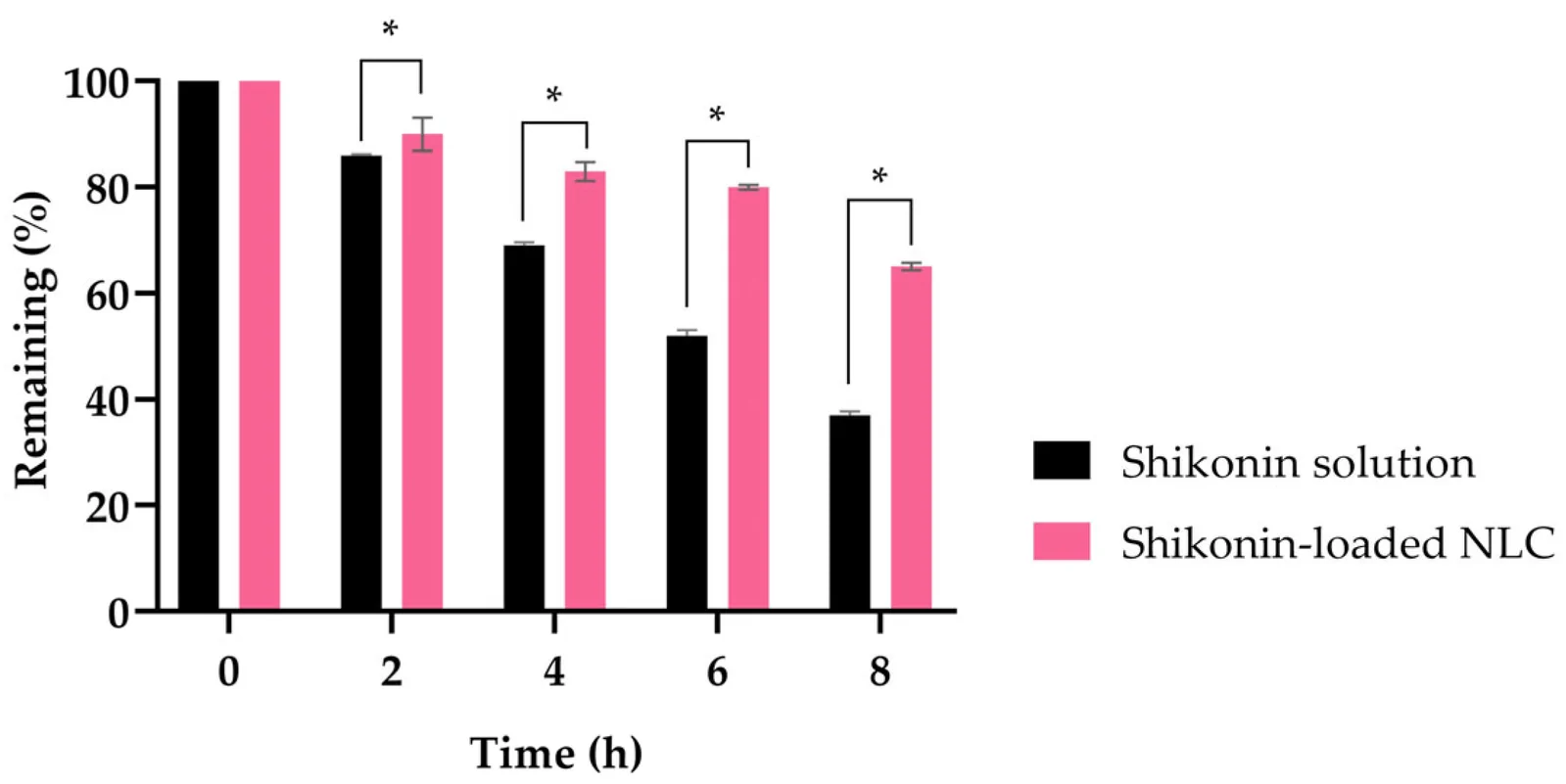
The remaining percentage of the shikonin in shikonin solution and shikonin-loaded NLC before and after photostability study. The values are expressed as mean ± SD (*n* = 3). Asterisk (*) indicates a statistically significant difference (*p* < 0.05) in multiple comparisons of samples, as determined by a two-way ANOVA.

The photoinstability of shikonin has been attributed to photo-oxidative degradation mechanisms. Cheng et al. (1995) reported that shikonin undergoes oxidation in the presence of molecular oxygen, primarily at the C-13 position of the allylic side chain [16]. This reaction is accompanied by double-bond migration and the formation of an allylic hydroperoxide intermediate. Therefore, prolonged light exposure may induce structural changes in shikonin, resulting in reduced chemical stability and potentially diminished biological activity. In the present study, the remaining percentage of the shikonin in solution was compared with shikonin-loaded NLC. The results are presented in Figure 9. Shikonin-loaded NLC exhibited greater photostability than the shikonin solution. The results clearly demonstrated that encapsulation within the NLC system significantly improved the photostability of shikonin compared with the free solution. The enhanced stability is primarily attributed to the ability of the lipid matrix to encapsulate shikonin and reduce its direct exposure to light and oxygen, thereby limiting photo-oxidative degradation. In addition, previous research have reported that the photostability of active compound can be enhanced through the incorporation of appropriate liquid lipids in lipid-based nanocarrier systems [22]. Structurally, NLC composed of solid and liquid lipids form an imperfect structure that prevents into ordered structures, thereby maintaining a high drug loading capacity and preventing the expulsion of actives [59]. Certain natural oils contain antioxidant constituents that can help reduce photo-oxidative degradation by scavenging reactive species generated during light exposure [60]. A key advantage in NLC development is the ability to select oil from a variety of liquid lipids, allowing the design of formulations optimized for each active compound. Sunflower oil has attracted considerable interest as a liquid lipid in pharmaceutical formulations. Arianto and Cindy (2019) reported that nanoemulsions containing sunflower oil exhibited higher sun protection factor (SPF) values than conventional emulsions during storage [61]. Furthermore, NLC formulations containing sunflower oil also increased the photostability and half-life of capsaicin and curcumin [22]. Therefore, sunflower oil was selected as the liquid lipid component in NLC formulations. Alpha-tocopherol is a well-known antioxidant and photoprotective agent found in sunflower oil that functions as a natural sunscreen, antioxidant, and UV-protective agent, helping to protect and inhibit the photodegradation of active compounds through the absorption and reflection of UV radiation [62,63].

### 3.5. In Vitro Release Study of Shikonin-Loaded NLC

**Figure 10:**
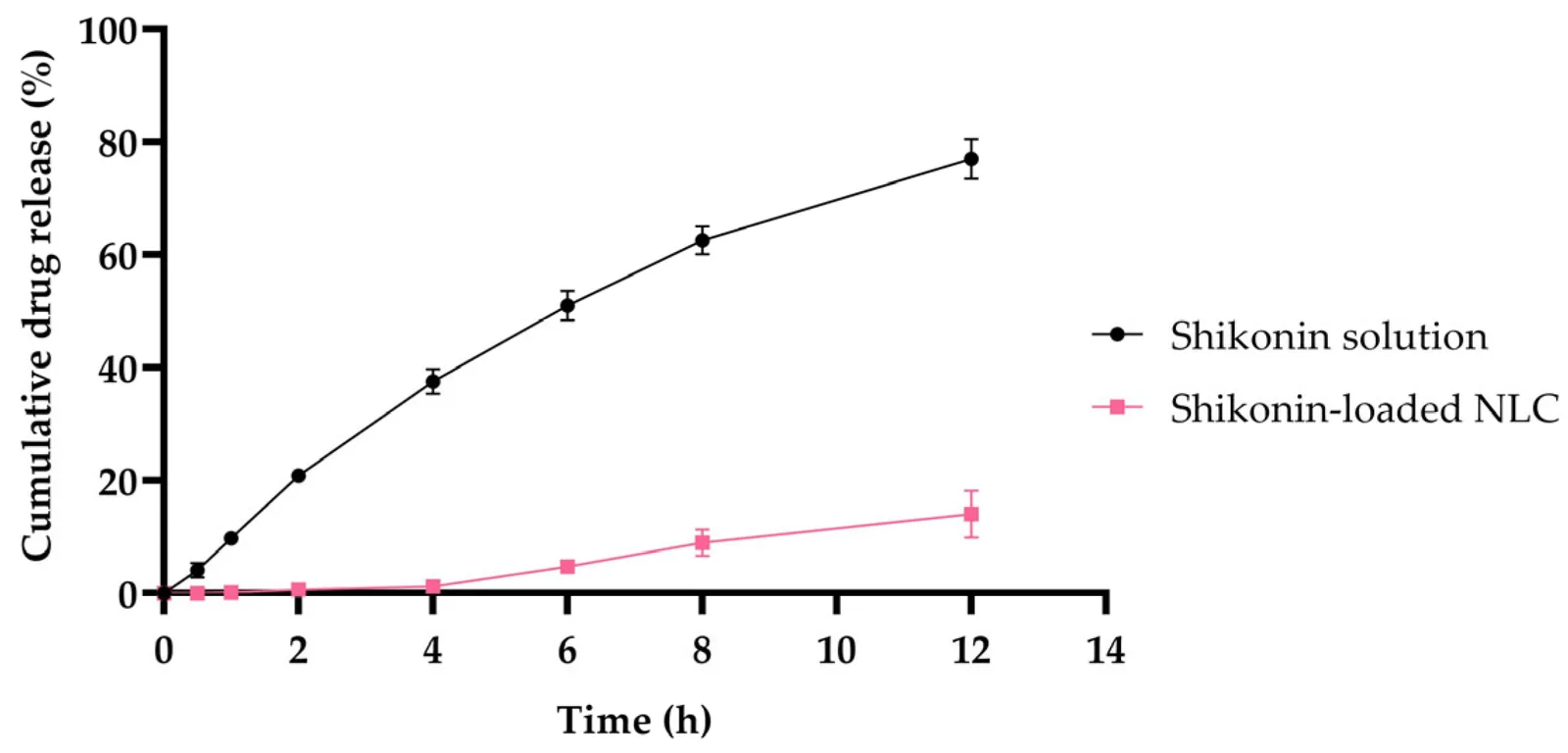
Comparative in vitro drug release profiles of shikonin in solution with shikonin-loaded NLC.

The release profiles of shikonin-loaded NLC compared to the shikonin in solution are presented in Figure 10. To maintain sink conditions throughout the release study, the volume of the release medium was adjusted to at least three times the volume required to completely dissolve the total amount of shikonin, thereby minimizing solubility-related limitations on drug release. The shikonin in solution exhibited rapid release behavior, reaching approximately 70% cumulative release within 12 h. The release kinetic model of shikonin solution was first-order model (R^2^ =0.998). In contrast, shikonin-loaded NLC showed slower release rate and lower cumulative release throughout the study period. This finding was further confirmed by kinetic modeling, which showed that the release data were best fitted by the Korsmeyer–Peppas model (R^2^ = 0.956), suggesting that drug release may involve a combination of diffusion and lipid matrix relaxation or erosion. The slow-release behavior observed in the NLC formulation suggests that the lipid nanoparticles effectively controlled the diffusion of shikonin from the lipid matrix [64,65,66]. The delayed release behavior of shikonin-loaded NLC could be attributed to the lipid matrix structure composed of solid lipid and liquid lipid, which efficiently entrapped the hydrophobic shikonin within the nanoparticles. Consequently, the shikonin requires diffusion through the lipid matrix before being released into the external medium, resulting in a slower release rate compared with the shikonin solution, in which the freely dissolved shikonin could readily diffuse across the dialysis membrane [67]. In addition, the release characteristic of shikonin-loaded NLC may provide advantages for dermal drug delivery applications by prolonging drug retention at the site of application, reducing dosing frequency [21], and enhancing the stability of the photosensitive shikonin by protecting it within the lipid matrix.

### 3.6. Development and Characterization of Hydrogel-Based Dermal Patches

The hydrogel-based dermal patches (P1–P5) were prepared with different concentrations of HA and CMC. All formulated patches were visually inspected to assess their physical appearance. In the preparation of hydrogel-based dermal patches, ethanol was selected as a volatile solvent to facilitate rapid evaporation and reduce drying time, while PG was used as a plasticizer to enhance the flexibility of the resulting patches. The patches were found to be homogeneous, smooth surfaces, colorless, flexible, soft, and a hydrogel-like texture in the hydrated state. Physicochemical and mechanical properties of hydrogel-based dermal patches are presented in Table 7. All formulations were evaluated by visual inspection for their physical appearance. The prepared patches exhibited similar characteristics, including a smooth surface, homogeneity, flexibility, and transparency, indicating successful formation of the hydrogel-based dermal patches from HA and CMC. The pH values of all formulations ranged from 6.7 to 6.8, which are within the acceptable range for topical application and compatible with the physiological pH of human skin [68,69]. The thickness of the hydrogel-based dermal patches ranged from 0.62 to 0.72 mm, while the patch weight ranged from 2.43 to 2.50 g. These values were consistent across formulations and within the acceptable range reported in previous studies on dermal patches [70], indicating good uniformity during preparation. The adhesive strength values ranged from 0.04 to 0.07 N. Although these values were relatively low. Therefore, the patch can be considered for dermal patch applications, particularly when used in combination with a backing layer to improve handling properties and skin attachment properties. The mechanical properties of the hydrogel-based dermal patches were evaluated through tensile strength and elongation at break. The tensile strength values ranged from 0.024 to 0.227 MPa, while the elongation at break ranged from 79.19 to 246.01%. Overall, all formulations exhibited acceptable mechanical properties for dermal patch applications, demonstrating an appropriate balance between strength and flexibility. In general, polymeric dermal patches exhibit tensile strength values below or more than 1 MPa and elongation at break values up to approximately 300%, depending on the polymer type, formulation composition, and testing conditions. The tensile strength increased progressively from P1 to P5, suggesting that increasing the proportion of CMC enhanced the mechanical strength of the hydrogel-based dermal patches. These findings are consistent with those reported by Kim et al. (2020) [40], which demonstrated that increasing the CMC concentration improved the mechanical properties of the patch. Formulation P5 significantly exhibited the highest tensile strength, which may be attributed to its higher CMC concentration, resulting in a denser polymeric network and stronger intermolecular interactions within the patch. In contrast, formulation P3 exhibited the significant highest elongation at break, indicating superior flexibility compared with the other formulations. This result suggests that the balanced ratio of HA and CMC in P3 formulation provided an optimal polymer network. However, further increases in CMC concentration, as observed in formulations P4 and P5, resulted in increased tensile strength but reduced elongation at break. This behavior indicated that the patches became mechanically stronger but less flexible when the proportion of CMC increased. Similar findings were reported by Ma et al. (2008) [71], who demonstrated that polymers with higher viscosity generally produce films with higher tensile strength but lower elongation at break. Furthermore, CMC might be promote intermolecular interactions within the polymer matrix, thereby increasing the rigidity of the network and contributing to the observed increase in tensile strength and reduction in elongation at break [72]. Therefore, formulations P3–P5 were selected for further incorporation studies with shikonin-loaded NLC.

### 3.7. Hydrogel-Based Dermal Patch Containing Shikonin-Loaded NLC

Importantly, the development of topical formulations for scar treatment requires a thorough understanding of the limitations and therapeutic requirements associated with scar management. Generally, scar tissue and newly healed wounds are often characterized by a fragile and vulnerable skin barrier. Therefore, maintaining adequate hydration and protecting the affected area from external contact, friction, and mechanical irritation are essential for preventing further tissue damage and supporting the healing process. Consequently, hydrogel-based dermal patches have emerged as an attractive topical delivery system because they can provide a moist wound environment, improve patient comfort, and prolong the residence time of active compounds on the skin. Furthermore, the incorporation of NLC into hydrogel matrices may further enhance drug stability, retention, and localized delivery. Therefore, the combination of NLC and hydrogel-based dermal patches represents a promising strategy for topical delivery that may be further developed for scar-related applications.

**Figure 11:**
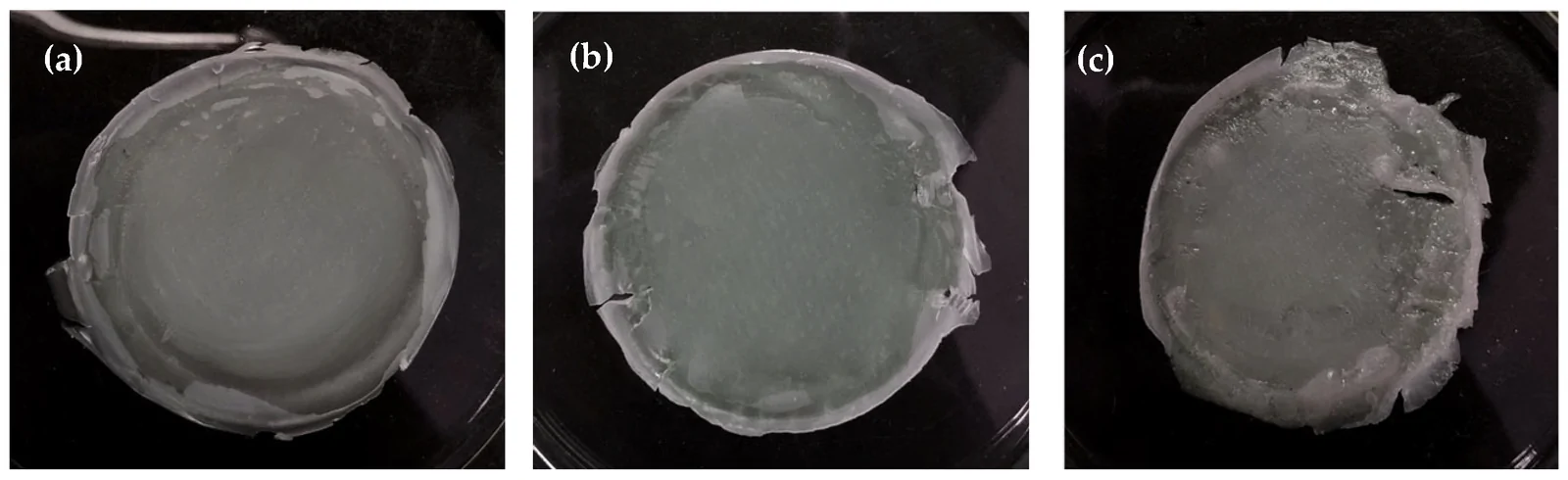
Physical appearance of dermal patch from the blank NLC-loaded hydrogel-based (P3) (**a**), P4 (**b**), P5 (**c**) at a 70:30 ratio (%*w*/*w*).

The hydrogel-based (P3–P5) formulations were incorporated with blank NLC at different ratios, and the results are shown in Table 8. The incorporation of blank NLC into the hydrogel-based formulations slightly increased the particle size, which may be attributed to interaction between the polymer chains and the nanoparticle surfaces [73,74]. Among all formulations, P3 exhibited the smallest particle size and relatively low PDI values, followed by P4 that indicates good particle dispersion and a relatively homogeneous size distribution. In contrast, P5 exhibited a large particle size and high PDI value, suggesting a broader particle size distribution and less uniform dispersion within the system. The zeta potential values remained negative for all formulations (−26.84 to −40.01 mV), indicating that the surface charge characteristics and colloidal stability of the NLC were greatly maintained after incorporation into the hydrogel system [75]. In addition, the proportion of CMC increased from P3 to P5, and a tendency toward increased viscosity of the formulation, larger particle size, higher PDI, and less negative zeta potential were observed. The physical characteristics of hydrogel-based dermal patches prepared from formulations P3–P5, as shown in Figure 11, demonstrated that formulation P3 at a 70:30% (*w*/*w*) ratio was the most suitable for hydrogel-based dermal patch preparation. This formulation produced an intact patch after peeling from the mold, with a homogeneous appearance, good stability, and no deformation. In contrast, formulations P4 and P5 at the same ratio showed fewer desirable characteristics. Furthermore, the 50:50 and 60:40% (*w*/*w*) ratios were unsuitable for hydrogel-based dermal patch preparation. Because higher of the blank NLC incorporated, the more interference with the drying process occurred, resulting in slower drying and failure to form appropriate patches. Although the 80:20 and 70:30% (*w*/*w*) ratios exhibited comparable physical characteristics, the 80:20% (*w*/*w*) ratio was not selected for further study because higher proportion of polymer matrix could reduce the drug-loading capacity of the formulation. This limitation may subsequently affect the efficiency of drug incorporation and drug delivery performance.

**Figure 12:**
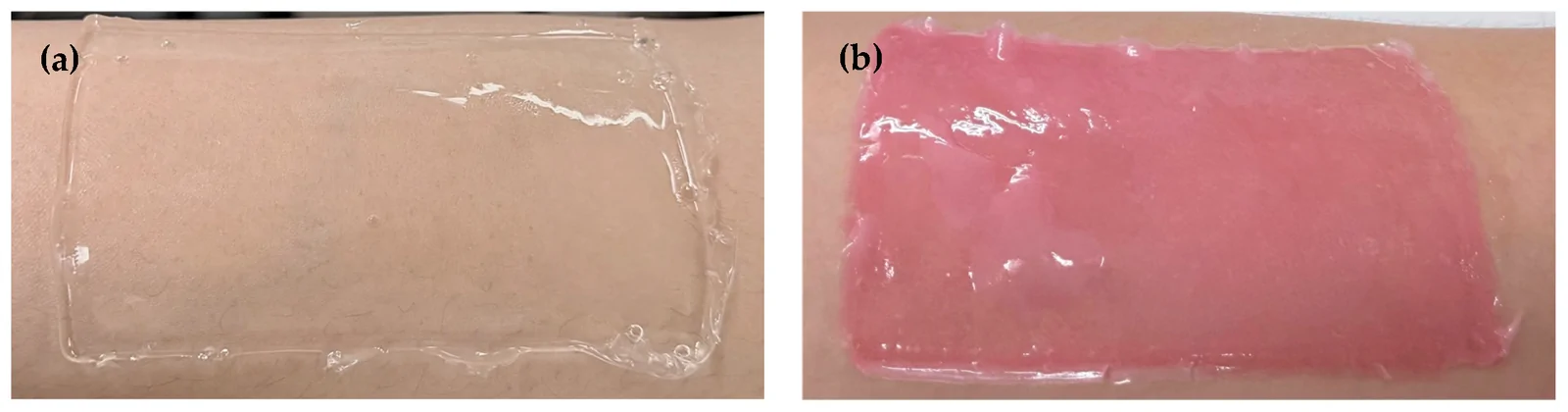
Physical appearance of hydrogel-based dermal patch (**a**) and hydrogel-based dermal patch containing shikonin-loaded NLC (**b**).

Therefore, the formulation P3 containing 70% hydrogel and 30% shikonin-loaded NLC (70:30, *w*/*w*) was selected as the optimal ratio for dermal patch preparation and subsequently cast in a silicone mold. The physical appearance of the hydrogel-based dermal patch containing shikonin-loaded NLC is presented in Figure 12. The hydrogel-based dermal patch exhibited a homogeneous and transparent appearance with a smooth surface, flexibility, and softness, which are typical characteristics of hydrogels in the hydrated state. After the incorporation of the shikonin-loaded NLC, the formulation exhibited a visual appearance in a pink color form of the shikonin compound, while maintaining uniformity throughout the matrix. In addition, it showed a slightly oily or glossy appearance. This characteristic is likely due to the presence of lipid components in the NLC formulation.

#### Characterization and Stability of Hydrogel-Based Dermal Patch Containing Shikonin-Loaded NLC

The hydrogel-based dermal patch containing shikonin-loaded NLC demonstrated good physical stability after storage at room temperature (30 °C) for one month. The results are shown in Table 9. The pH value, color, and adhesive strength of the hydrogel-based dermal patch containing shikonin-loaded NLC did not change during the storage period. The patch remained pink in color, indicating that shikonin retained its characteristic appearance. The pH values remained relatively constant (7.2–7.1), demonstrating the stability of the formulation and suggesting compatibility with the human skin. Although the hydrogel-based dermal patch containing shikonin-loaded NLC exhibited a slightly higher pH (7.20 ± 0.02) than the unloaded hydrogel-based dermal patch (6.70 ± 0.01), this difference was statistically significant (*p* < 0.05). Nevertheless, both formulations exhibited pH values close to neutrality, suggesting that the observed difference is unlikely to substantially affect skin compatibility or increase the risk of skin irritation. Slight increases in thickness and weight (not more than 10%) after storage may be attributed to moisture absorption by the nature of the hydrogel system. Hydrophilic polymers can absorb and retain moisture from the environment, leading to minor variations in the physical properties of the patches during storage. However, these changes were minimal and did not affect the physical integrity of the patch. In addition, the developed hydrogel-based dermal patch exhibited good flexibility and structural integrity throughout handling and storage without visible cracking, fragmentation, or deformation. Overall, the results indicate that the hydrogel-based dermal patch containing shikonin-loaded NLC possessed satisfactory physical stability under the tested storage condition.

### 3.8. Membrane Retention and Permeation Study

**Figure 13:**
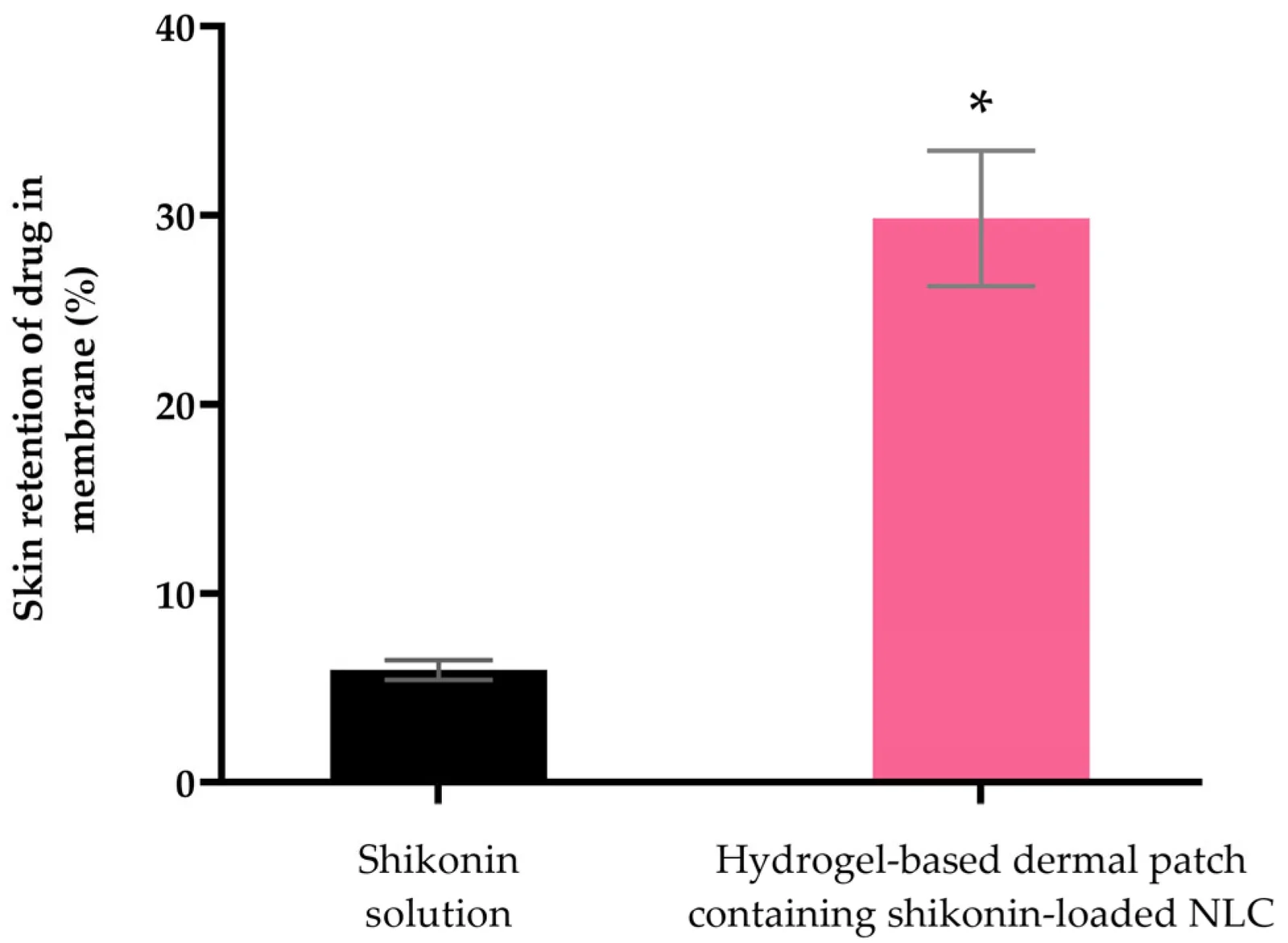
Percentage membrane retention of shikonin from shikonin in solution and hydrogel-based dermal patch containing shikonin-loaded NLC. Asterisks (*) indicate statistically significant difference between both formulations (*p* < 0.05), as determined by an independent samples *t*-test.

Skin permeation of the hydrogel-based dermal patch containing shikonin-loaded NLC was evaluated by Franz diffusion cells using Strat-M^®^ synthetic membrane. The Strat-M^®^ membrane possesses a multilayered structure designed to mimic the permeability characteristics of human skin. Its layered architecture resembles the barrier properties of the epidermis, dermis, and subcutaneous tissue, making it a widely accepted synthetic model for in vitro skin permeation studies [76]. No detectable shikonin was observed in the receptor compartment throughout the study period. This finding may be attributed to the slow-release behavior of the NLC system and the retention of shikonin within the Strat-M^®^ membrane rather than complete diffusion across the membrane barrier. After 24 h of skin permeation study, the membrane was extracted with methanol to determine the retained amount of shikonin. The results of membrane retention are presented in Figure 13. The hydrogel-based dermal patch containing shikonin-loaded NLC exhibited significantly greater drug retention in the membrane than shikonin in solution. These findings indicated that the incorporation of shikonin into the NLC can enhance membrane deposition and retention of the compound in the membrane. Shikonin is a lipophilic compound, which may promote its partitioning into the lipid components of the skin and accumulation within the epidermal layers. Furthermore, shikonin was encapsulated within the NLC system with a small particle size (less than 150 nm), which enhanced skin penetration by enabling intimate contact between the nanoparticles and the outermost skin layer. This interaction may increase the fluidity of the stratum corneum lipid matrix, thereby facilitating deeper penetration of the particles into the skin layers [77]. Nanoparticle skin interactions are highly dependent on the particle size, shape, and functional groups on the nanoparticle surface, and hydrophobicity/hydrophilicity, all of which can influence nanoparticle adhesion and cellular uptake [78]. The hydrogel-based dermal patch system may contribute to enhanced skin penetration by maintaining close and prolonging contact between the formulation and the skin surface area [79]. In addition, the occlusive properties of the hydrogel-based dermal patch could increase skin hydration, leading to swelling and loosening of the stratum corneum structure, which facilitates drug diffusion into the deeper skin layers [80]. In combination with the dermal patch and the NLC formulation, this prolonged residence time on the skin may further promote shikonin accumulation within the membrane layers.

## 4. Conclusions

This study demonstrated the successful development and optimization of shikonin-loaded NLC and incorporated into a hydrogel-based dermal patch. The optimized NLC exhibited desirable physicochemical characteristics, including nanoscale particle size, narrow size distribution, high entrapment efficiency, and good physical stability. Notably, encapsulation of shikonin within the NLC significantly improved its photostability and provided slow drug release behavior. The incorporation of shikonin-loaded NLC into the hydrogel matrix resulted in a flexible, homogeneous, and physically stable dermal patch suitable for further development for topical application. The developed hydrogel-based system effectively modulated shikonin release and promoted prolonged retention of the active compound within the membrane, which may be ben eficial for a localized topical product. These findings demonstrate the potential of lipid-based nanocarriers to overcome the physicochemical limitations of photosensitive compounds to enhance topical drug delivery performance. Overall, the hydrogel-based dermal patch containing shikonin-loaded NLC represents a promising biomaterial platform for the localized delivery of shikonin. The integration of enhanced photostability, slow release, and increased membrane retention provides a foundation for further investigation of this system topical pharmaceutical applications. For future study, biological activity, long-term stability, and clinical study of the hydrogel-based dermal patch containing shikonin-loaded NLC should be evaluated for further establish its therapeutic potential.

## Figures and Tables

**Table 1:** Composition of blank NLC formulations prepared with different lipid concentrations.

Ingredient	Quantity (% *w*/*w*)			
	F1	F2	F3	F4
Glyceryl distearate	3.00	6.00	9.00	12.00
Sunflower oil	2.00	4.00	6.00	8.00
Sorbitan oleate	18.88	18.88	18.88	18.88
Polysorbate 80	1.12	1.12	1.12	1.12
DI water	q.s.100	q.s.100	q.s.100	q.s.100

**Table 2:** Composition of blank NLC formulations prepared with different mixed surfactant ratios.

Ingredient	Quantity (% *w*/*w*)						
	A	B	C	D	E	F	G
Glyceryl distearate	3.00	3.00	3.00	3.00	3.00	3.00	3.00
Sunflower oil	2.00	2.00	2.00	2.00	2.00	2.00	2.00
Sorbitan oleate	10.00	13.33	15.00	16.00	6.67	5.00	4.00
Polysorbate 80	10.00	6.67	5.00	4.00	13.33	15.00	16.00
DI water	q.s.100	q.s.100	q.s.100	q.s.100	q.s.100	q.s.100	q.s.100

**Table 3:** Compositions of hydrogel-based dermal patches.

Formulation			Quantity (% *w*/*w*)		
	HA	CMC	Ethanol	PG	DI Water
P1	0.85	0.15	15	5	q.s.100
P2	0.65	0.35	15	5	q.s.100
P3	0.50	0.50	15	5	q.s.100
P4	0.35	0.65	15	5	q.s.100
P5	0.15	0.85	15	5	q.s.100

**Table 4:** Ratios of hydrogel to blank NLC formulation.

Formulation	Ratios (% *w*/*w*)	
	Hydrogel	Blank NLC
1	50	50
2	60	40
3	70	30
4	80	20

**Table 5:** Particle size, PDI, and zeta potential of blank NLC before and after the stability test. The data are expressed as mean ± SD (*n* = 3).

Parameters	Formulation F1E	
	Day 0	Day 30
Particle size (nm)	78.41 ± 0.01	73.73 ± 0.01 *
PDI	0.27 ± 0.01	0.26 ± 0.01
Zeta potential (mV)	−38.81 ± 0.05	−33.64 ± 0.03 *

Asterisk (*) indicates a statistically significant difference (*p* < 0.05) between day 0 and day 30, as determined by a paired sample *t*-test.

**Table 6:** Particle size, PDI, and zeta potential of shikonin-loaded NLC before and after the stability test. The data are expressed as mean ± SD (*n* = 3).

Shikonin-Loaded NLC					
Parameters	Day 0	Day 30			
		RT	H/C	4 °C	45 °C
Particle size (nm)	104.94 ± 0.01	107.23 ± 0.03 *	98.04 ± 0.03 *	94.95 ± 0.01 *	111.21 ± 0.01 *
PDI	0.15 ± 0.00	0.22 ± 0.00 *	0.16 ± 0.00	0.22 ± 0.00 *	0.13 ± 0.00 *
Zeta potential (mV)	−34.34 ± 0.02	−33.80 ± 0.02 *	−32.50 ± 0.02 *	−23.51 ± 0.01 *	−30.43 ± 0.02 *
Drug recovery (%)	100 ± 0.00	87.46 ± 9.61	77.71 ± 4.99 *	93.66 ± 3.26	85.14 ± 1.14 *

Asterisk (*) indicates a statistically significant difference (*p* < 0.05) between day 0 and day 30 at each storage condition, as determined by a paired sample *t*-test.

**Table 7:** Physicochemical and mechanical properties of hydrogel-based dermal patches.

Formulation	pH	Thickness (mm)	Weight (g)	Adhesive Strength (N)	Tensile Strength (MPa)	Elongation at Break (%)
P1	6.7	0.70 ± 0.02	2.50 ± 0.02	0.04 ± 0.01	0.024 ± 0.006 ^b^	79.19 ± 7.36 ^e^
P2	6.7	0.72 ± 0.10	2.48 ± 0.01	0.05 ± 0.01	0.028 ± 0.009 ^b^	102.25 ± 7.39 ^d^
P3	6.7	0.68 ± 0.06	2.49 ± 0.03	0.06 ± 0.01	0.056 ± 0.001 ^b^	246.01 ± 3.33 ^a^
P4	6.8	0.67 ± 0.02	2.43 ± 0.01	0.06 ± 0.00	0.085 ± 0.040 ^b^	193.40 ± 5.76 ^b^
P5	6.8	0.62 ± 0.12	2.43 ± 0.02	0.07 ± 0.01	0.227 ± 0.046 ^a^	170.98 ± 6.21 ^c^

Different letters (a–e) indicate statistically significant differences among samples (*p* < 0.05), as determined by one-way ANOVA followed by Tukey post hoc test.

**Table 8:** Particle size, PDI, and zeta potential of hydrogel-based dermal patch containing blank NLC. The data are expressed as mean ± SD (*n* = 3).

Formulation	Ratios (% *w*/*w*)		Characterizations		
	Hydrogel	Blank NLC	Particle Size (nm)	PDI	Zeta Potential (mV)
Patch P3	50	50	79.97 ± 0.38	0.29 ± 0.01	−38.38 ± 0.02
	60	40	84.97 ± 0.01	0.30 ± 0.02	−37.80 ± 0.01
	70	30	89.21 ± 0.02	0.35 ± 0.02	−40.01 ± 0.02
	80	20	90.46 ± 0.01	0.36 ± 0.01	−37.53 ± 0.01
Patch P4	50	50	90.46 ± 0.01	0.28 ± 0.00	−34.15 ± 0.01
	60	40	86.91 ± 0.01	0.27 ± 0.00	−37.54 ± 0.02
	70	30	90.61 ± 0.03	0.28 ± 0.00	−33.89 ± 0.02
	80	20	104.60 ± 0.01	0.42 ± 0.00	−38.99 ± 0.01
Patch P5	50	50	92.07 ± 0.01	0.33 ± 0.00	−31.55 ± 0.01
	60	40	121.27 ± 0.01	0.42 ± 0.00	−36.24 ± 0.01
	70	30	115.37 ± 0.02	0.41 ± 0.00	−30.13 ± 0.02
	80	20	115.94 ± 0.02	0.42 ± 0.00	−26.84 ± 0.01

**Table 9:** Characteristics of hydrogel-based dermal patch containing shikonin-loaded NLC before and after stability test. The data are expressed as mean ± SD (*n* = 3).

Time	Characterizations				
	pH (Solution)	Color	Thickness (mm)	Weight (g)	Adhesive Strength (N)
Day 0	7.2	pink	0.69 ± 0.02	2.35 ± 0.01	0.06 ± 0.01
Day 30	7.1	pink	0.75 ± 0.02 *	1.56 ± 0.03 *	0.07 ± 0.00

Asterisk (*) indicates a statistically significant difference (*p* < 0.05) between day 0 and day 30, as determined by a paired sample *t*-test.

## Data Availability

The data supporting the findings of this study are available within this published article.

## Abbreviations

The following abbreviations are used in this manuscript:

ACN	Acetonitrile
CMC	Carboxymethyl cellulose
DMSO	Dimethyl sulfoxide
ECM	Extracellular matrix
HA	Hyaluronic acid
HPLC	High-performance liquid chromatography
Log P	Logarithm of the partition coefficient
NLC	Nanostructured lipid carriers
PBS	Phosphate-buffered saline
PEG400	Polyethylene glycol 400
PDI	Polydispersity index
PG	Propylene Glycol
R_t_	Retention time
SC	Stratum corneum
SD	Standard deviation
SLN	Solid lipid nanoparticle
SPF	Sun protection factor
TEM	Transmission electron microscope
THF	Tetrahydrofuran
UV	Ultraviolet
%EE	Percentage entrapment efficiency
